# Targeted Drug Delivery Strategies in Overcoming Antimicrobial Resistance: Advances and Future Directions

**DOI:** 10.3390/pharmaceutics17111426

**Published:** 2025-11-04

**Authors:** Ohoud M. Alidriss, Hamood AlSudais, Ohoud S. Alhumaidan, Haifa D. Altwaijry, Afnan Bakhsh, Yasir Almuhanna, Zeina S. Alkudmani, Ibrahim A. Alqarni, Daheeya Alenazi, Alanoud T. Aljasham, Yahya F. Jamous

**Affiliations:** 1 Department of Clinical Laboratory Sciences, College of Applied Medical Sciences, King Saud University, Riyadh 12372, Saudi Arabia; halsudais@ksu.edu.sa (H.A.); oalhumaidan@ksu.edu.sa (O.S.A.); haaltwaijry@ksu.edu.sa (H.D.A.); afbakhsh@ksu.edu.sa (A.B.); zalkudmani@ksu.edu.sa (Z.S.A.); daalenazi@ksu.edu.sa (D.A.); aaljasham@ksu.edu.sa (A.T.A.); 2 Department of Clinical Laboratory Sciences, College of Applied Medical Sciences, Shaqra University, Shaqra 11961, Saudi Arabia; yalmuhanna@su.edu.sa; 3 Department of Physiology, College of Medicine, King Saud University, Riyadh 11421, Saudi Arabia; ialqarni@ksu.edu.sa; 4 Wellness and Preventive Medicine Institute, Health Sector, King Abdulaziz City for Science and Technology, Riyadh 11442, Saudi Arabia

**Keywords:** antimicrobial resistance, multidrug resistant, targeted drug delivery, nanoparticulate carrier systems, stimuli-responsive systems, antibody–drug conjugates, CRISPR-Cas systems

## Abstract

Antimicrobial resistance (AMR) is a present, pressing global public health crisis associated with rising morbidity and mortality rates due to previously curable infectious disease. Targeted drug delivery is an important approach to address AMR due to its ability to improve the therapeutic performance of antibiotics without leading to any adverse effects or organ toxicities. In this review we explore molecular mechanisms of AMR and drawbacks of conventional antibiotic therapies and discuss unique drug delivery approaches to compensate these. Nanoparticulate carrier systems, stimuli-responsive systems, antibody–drug conjugates, and CRISPR-Cas systems are some of the carrier method designs that are promising for tackling hard to treat infections related to pathogenic strains and biofilms due to their features. Many of these are among the most significant advances in the field. However, there are many challenges to be overcome, with biological limitations, scaling and regulatory challenges, etc., before they can be employed in commercial applications. Materials are being developed, and an approach standardized and applicable to future work is in development to improve the efficiency of targeted delivery systems. Controlled drug delivery, which could be the answer to an increasing AMR problem, will not only help in alerting awareness among individuals but will also help in prolonging the activity of antibiotics by providing synergistic interdisciplinary solutions. This review emphasizes the complementary role of targeted drug delivery in transitioning from laboratory investigations to clinical therapy. It addresses underrepresented aspects, including new materials, scalability, regulatory considerations, and ethical implications, while offering a roadmap for translating innovations into next-generation antimicrobials.

## 1. Introduction

Antimicrobial resistance (AMR) is when bacteria, viruses, fungi, and parasites change over time and no longer respond to drugs such as antibiotics and antivirals, making infections harder to treat and increasing costs as well as the risk of disease spread, severe illness, and death. The world is wrestling with the terrifying prospect of an increasingly antimicrobial-resistant world, in which bacteria become immune to some of the most potent weapons we have developed to combat infection. The emergence of MDR organisms is the result of this resistance mechanism, which exerts serious threats to public health infrastructures, healthcare delivery, systems, and the economic systems globally. A valuable strategy for this priority issue is a thorough understanding of AMR mechanisms and historical observations on the consequences of AMR, including current measures to overcome it. Microorganisms have multiple routes to becoming resistant. Resistance to the action of antibiotics by genetic mutations of drug targets or the development of efflux systems with specific characteristics is caused by mutations [1]. Bacteria can already do this efficiently, with horizontal gene transfer allowing resistance genes to be snapped up and rapidly distributed throughout bacterial populations.

**Figure 1:**
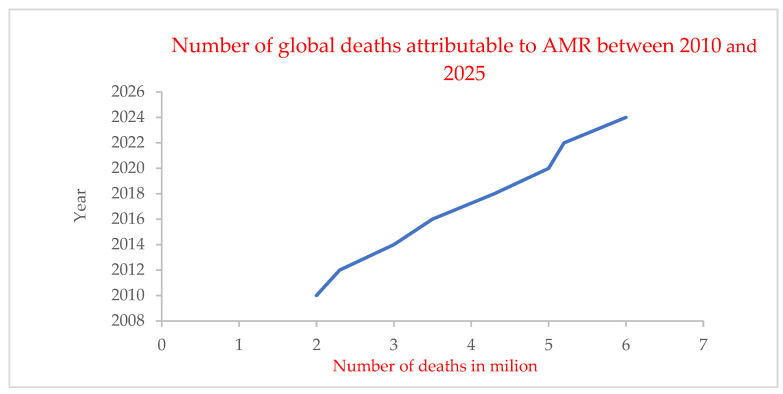
Global deaths due to AMR over the years. The figure estimates that >6 million people will die of AMR in 2025 [2].

AMR is a global challenge that undermines the effectiveness of antibiotics and the treatment of infections, resulting in an estimated 4.95 million deaths worldwide in 2019 (Figure 1). The data demonstrate a consistent and substantial increase in AMR-related deaths, rising from approximately 2 million in 2010 to nearly 6 million by 2025 [2]. Moreover, 1.27 billion deaths are a result of lower respiratory infections, and 1.5 billion occurred as a result of inappropriate, extended, non-reducing prescriptions and misuse; systemic abuse and overuse; and continued subtherapeutic drug exposure [3,4,5]. AMR-related deaths are estimated to have surged to and surpassed the levels predicted for 2050, with over 10 million deaths/year attributed to resistant infections. The overall economic consequences of AMR, on the other hand, are estimated to exceed USD 100 trillion when considering the direct healthcare costs and loss in workforce productivity [6,7]. Overutilization of antibiotics is a major force driving the emergence of resistance, affording non-targeted bacterial sectors exposure to selection, circumventing the more extended diffusion of resistance at random (and so forth), and freely exchanging and slow (and in the case of human-targeted bacteria, frequently higher-stakes) genetic exchange [8,9]. For example, long-term exposure to sub-minimum inhibitory concentrations (sub-MICs) can provide an environment for the development of drug-resistant mutations and for horizontal gene transfers of resistance, which could render even non-pathogenic commensals resistant [10,11].

Control of chronic infections represents a major challenge because chronic infection prevalence is rising due to the spread of AMR. While antibiotics are in widespread use, they tend to worsen the situation as the microbial population is selectively pressured to select for resistance [8]. As priority pathogens of the World Health Organization (WHO), *E. coli* and *K. pneumoniae* have generated resistance to beta-lactams, colistin, and other first-line antibiotics [12]. These organisms also generate different types of ESBL, which, in contrast, restrict the drug options to control infection [13]. The first evidence of resistance to more than one antibiotic was reported in the late 1950s and early 1960s with enteric bacterial organisms such as Shigella, Salmonella, and *E. coli* [14,15]. This resistance has now also been extended to Gram-positive and Gram-negative pathogens, and since then, many of the antibiotics on hand have not been effective [16]. The infections are particularly challenging in ICUs and have been correlated with prolonged durations of hospitalization, therapeutic failures, and increased morbidity [17,18,19,20]. Furthermore, there is an issue in the use of nonspecific antibiotics, which are used in the treatment of biofilm-related infections. They result from bacteria secreting an extracellular matrix composed of polymeric substances such as polysaccharides; from it, a hydrogel is created, which acts as a diffusion barrier. This defines the prevention of the penetration of an antibiotic into the microorganism, as a result of which pathogens can tolerate the usual course of treatment. This results in the need for considerably higher doses to achieve an efficacious effect [21]. Antibiotics and other broad-spectrum antibiotics usually cannot penetrate and kill bacterial biofilm populations, resulting in widespread occurrence of chronic infections, preferentially in medical implants and devices [22,23]. This has resulted in a global public health problem of an increase in inpatient morbidity, mortality, and health care costs. To increase therapeutic success and minimize healthcare burden, there is a pressing need to develop a means of antibiotic delivery that targets individual resistance mechanisms.

Antimicrobial stewardship programs (ASPs) are required to improve antibiotic use and prevent antibiotic resistance [1]. Efforts to investigate the use of phages and monoclonals deserve special mention from a scientific point of view and may provide optimism for new medical approaches that can bypass conventional resistance strategies [6]. The One Health approach requires coordinated efforts between human, animal, and environmental health to address AMR. There are various approaches used to counteract AMR, including the discovery of new antibiotics, the maximization of existing therapies, and stewardship for better use of antimicrobials with improved responsibility. But new antibiotics are costly and time consuming to bring to market, and following through on stewardship programs can be difficult. Emerging drug delivery strategies have the potential to enhance overall antimicrobial therapy efficacy, minimize resistance selection, and exploit a therapeutic approach against drug-resistant strains. A targeted nanomedicine (TPNs/ICG-cRGD) selectively targets activated platelets, enables stimuli-responsive therapy, has photothermal and anti-inflammatory properties, and combines multiple therapeutic mechanisms in one platform, which can overcome microbial resistance barriers, just as they overcome physiological barriers in thrombosis. These multifunctional nanocarriers can be engineered to recognize bacterial membranes, infection microenvironments, or components of biofilms [24]. A Small Molecule–Drug Conjugate (SMDC) has been engineered with an Fc-protein carrier (Fc-EC140) to overcome pharmacokinetic limitations, improving circulation time, target selectivity, and therapeutic efficacy. Therefore, by conjugating antibiotics or antimicrobial agents to targeting ligands and extending their circulation through Fc or polymer conjugation, it becomes possible to achieve prolonged drug exposure at infection sites. This technique could promote bacterial elimination, minimize the development of resistance, and reduce required dosing frequency [25]. It is expected that such systems will offer new prospects for innovative therapy against resistant infections and a solution to the threat to public health caused by antimicrobial resistance [8].

## 2. Mechanisms of AMR

### 2.1. Genetic and Biochemical Mechanisms

**Figure 2:**
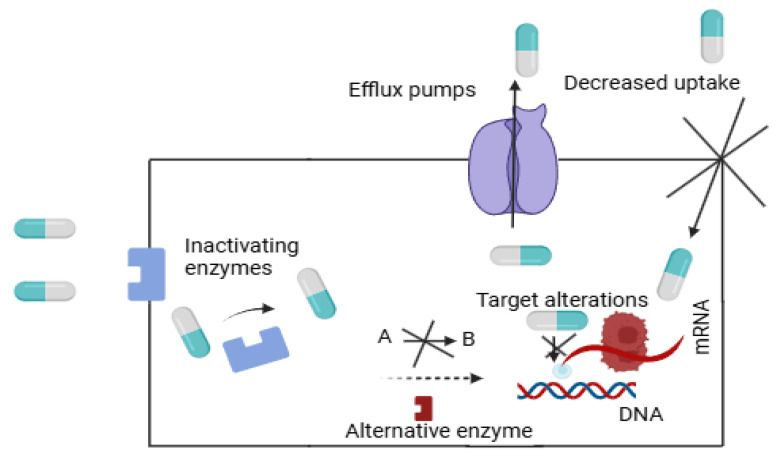
Mechanisms of bacterial resistance to antibiotics. As shown in Figure, one common mechanism is related to the use of efflux pumps, which are specialized proteins embedded in the bacterial cell membrane and actively extrude antibiotics from the cell, thus decreasing their intracellular effective concentration. A second strategy, “decreased uptake”, involves bacteria restricting the permeability of their cell membranes so that antibiotics cannot pass through. In addition, bacteria produce inactivating enzymes that chemically modify or break down antibiotics, such as β-lactam antibiotics (like penicillin), through the action of β-lactamases. It can also arise with target alterations, where bacteria modify the molecular structures of the enzymes, ribosomes, or DNA targeted by antibiotics so that the molecules cannot bind to the drugs and exert their effect. Bacteria may finally have an alternative enzyme to use, designed to bypass metabolic pathways that antibiotics prevent them from using, thereby allowing them to function without being blocked by the drug (image created with BioRender.com).

Efflux pumps are proteins located on the borders within bacteria, and they assist in bacterial survival as well as antibiotic resistance. These membrane proteins drastically reduce the concentration of antibiotics inside cells by actively pushing various substances, including antibiotics, out of the cell (Figure 2). With the decreased concentration of drugs, lower susceptible concentrations are maintained in their vicinity, with increased chances of survival for bacteria which exhibit active efflux pumps. Efflux pumps, activated by active transport mechanisms, need energy to pump antibiotics out of cells, leading them to be less useful [26,27]. Biofilm emergence is encouraged by efflux pumps operating in the bacterium *Staphylococcus aureus*. Moreover, bacterial combination works on the principles of biofilm, providing it with a guarded shield from immune responses and most likely rendering antibiotics useless, making treatment more challenging and management laborious [27]. Regarding *Helicobacter pylori*, antibiotics are hard to use in treatment because the mechanisms of resistance depend mainly on how these pumps function [26]. The Mycobacterium abscessus pathogen demonstrates intrinsic resistance primarily through its effective antibiotic efflux processes [28]. Among Gram-negative bacteria resistance–nodulation–division (RND) efflux pumps display primacy in furthering multidrug resistance because they respond to environmental influences [29]. Research evidence demonstrates how targeting efflux pumps would facilitate existing antibiotics, yet the presence of these pumps continues to spur the problem of antibiotic resistance. The proposed method shows prospective value as an effective strategy against bacterial strains that have become drug-resistant [30]. To summarize, efflux pumps reduce intracellular antibiotic concentrations, thereby reducing drug efficacy and promoting multidrug resistance in various bacterial species.

Beta-lactamases (inactivating enzymes) are a group of enzymes produced by bacteria that confer resistance to beta-lactam antibiotics by hydrolyzing the beta-lactam ring common to penicillins, cephalosporins, and carbapenems (as shown in Figure 2). As a result, these antibiotics are not able to block their primary targets called penicillin-binding proteins (PBPs), which are the key enzymes in bacterial cell wall synthesis due to this enzymatic cleavage. Beta-lactamases are divided into four different classes (A–D) according to their molecular structure and behavior. One of the most important aspects of Gram-negative bacteria is MBLs or Class B beta-lactamase genes due to their extreme capability for wide antibiotic resistance within beta-lactam antibiotics due to a vast variety of beta-lactams being destroyed, as observed by De Souza et al. [31]. When enzymes activate this process, an amide bond inside the beta-lactam ring becomes breakable, leading to the breakdown of antibiotics [32].

Beta-lactamases have established themselves as major resistance factors because beta-lactamase genes are spreading widely across resistant bacterial strains, including members of the ESKAPEE pathogen group. Under antibiotic selection pressure the evolution of these genes demonstrates an advantage [31]. Beta-lactamases evolve through environmental pressures according to studies, which demonstrates that they might gain stronger resistance mechanisms [33]. The effectiveness of beta-lactamase inhibitors faces significant barriers when the target is the MBL group of pathogens. Initial research on beta-lactam inhibitors provides strong results against serine beta-lactamases yet weak results against MBLs, so additional studies must improve inhibitor development [34]. Beta-lactamases represent the principal resistance mechanism, yet efflux pumps and target site mutations combine to make antimicrobial resistance extremely difficult to combat. Briefly, beta-lactamases enzymatically degrade beta-lactam antibiotics and are therefore one of the most widespread and clinically relevant resistance mechanisms.

In cell wall target modification, bacterial cells require cell wall synthesis events, PBPs, and peptidoglycan layer components to maintain their shape (Figure 2). PBPs work to create essential peptidoglycan connections that stabilize bacteria within their cell walls while serving as a fundamental requirement for peptidoglycan synthesis completion [35,36]. Cell wall stability depends on enzyme-mediated peptide chain activation through D,D-transpeptidases to create essential cross-links in the structure of the peptidoglycan. Beta-lactams together with glycopeptides represent the primary antibacterial mechanisms that impact cell wall synthesis during the process of cell division. The antibiotic group of beta-lactams functions by imitating PBP substrate activity to establish irreversible bonds with the proteins, leading to cell wall synthesis failures and subsequent cell death [35,36]. Glycopeptides, including vancomycin, disrupt cell wall synthesis by binding to the D-Ala-D-Ala terminus while blocking peptidoglycan precursor addition to the developing cell wall [37].

Biofilm is a complex community of microorganisms that is embedded in a self-secreted EPSs (extracellular polymeric substances) and attaches to either biotic or abiotic surfaces. The EPS matrix, consisting primarily of polysaccharides, proteins, lipids, and eDNA, acts as a physical barrier that restricts the diffusion of antibiotics and host immune cells in bacterial cells. This structural complexity results in poor penetration of antimicrobials into the armor and ensures the survival of bacteria within the deeper layers of the biofilm, where drug concentrations could be lethal.

The development of biofilm-associated infections depends on persister cells, which are “inert” members of the sessile bacterial population and are highly tolerant to antibiotics. Unlike resistant mutants, persisters are not the result of genetic change but rather a transient, non-dividing state that is assumed by cells under stress. This state of dormancy is characterized by these cells being metabolically dormant, and the majority of antibiotics that kill predominantly actively dividing cells do not kill these cells. They may repopulate the lung upon treatment withdrawal and give rise to chronic or recurrent infection [38]. Dense, robust biofilm can also increase distributions of HGT through transformation, transduction, and conjugation. The physical isolation of the cells in the biofilm enhances the spread of plasmids and determinants of resistance, enabling that resistance to antimicrobials to be transferred to the bacteria that have not yet acquired it. Mobile genetic elements like integrons and transposons are also frequently found in biofilm and contribute to the increasing risk of MDR [39,40].

Many genetic factors regulate biofilm development and maturation. Quorum sensing (QS) is a bacterial communication system for regulating intercellular gene expression and functions according to a cell density ratio. In biofilms, QS controls EPS production, exudation of VF, and dispersion. Small regulatory RNAs (sRNAs) play a role in posttranscriptional regulation of primary genetic signaling pathways but also participate in stress processes, metabolic pathways, and the expression of resistance genes [41]. QS and sRNAs act in synergy in regulating the group work of the biofilm, thus enhancing its adaptive and antimicrobial resistance policy. The signaling molecule c-di-GMP activates biofilm by regulating fimbrial genes and motility-related genes [42]. While *E. coli* depends on type 1 fimbriae for rapid early onset of biofilm development, *M. smegmatis* relies on GroEL1 and Lsr2 for intercellular cohesion and for the architectural stability of the biofilm [43,44]. A change in the expression of different genes occurs in suitable conditions of biofilm development; that is why the genetic regulation is complex and different during the life cycle of biofilm [43,44]. Environmental conditions that are in a dynamic balance with genetic determinants dictate the development of a biofilm as a whole. In summary, biofilms produce a protective microenvironment that enhances resistance to antibiotics by way of physical exclusion, metabolic dormancy, and gene transfer.

Genomic mutations are well known to be involved in emergence and persistence of AMR, particularly in selective antibiotic pressure (Figure 2). Such mutations can be spontaneous or induced by the environment and may include changes in drug target/tentacles, membrane permeability, or enzyme function. Modification of the antibiotic target, such as by acquisition of point mutations in the ribosomal protein genes of tetracyclines; in the DNA gyrase and RNA polymerase genes of fluoroquinolones and rifamycins, respectively; or in the PBPs of beta-lactams is a documented resistance mechanism in bacteria. BTK mutations result in protein geometry alterations in the binding site and drug resistance to ibrutinib therapy [45]. Furthermore, changes in regulatory genes may overexpress efflux pumps or downregulate porins, resulting in decreased antibiotic accumulation within bacterial cells. Clonal or horizontal genetic transfer is the process favoring the persistence and fixation of resistant mutants. Not only are such mutations potentially cross-resistance determinants to unrelated antimicrobial families, but they are also likely to enhance the complexity in management of treatment options. The genetic mutations are spontaneous or selected due to the use of drugs that allow bacteria to evolve to avoid others. They are always responsible for resistance-specific, broad-spectrum-specific, and broad-spectrum resistance, and the surveillance of mutations is imperative in the control strategies of AMR.

Resistance mutations and bacterial targets can nowadays be predicted through computational biology and machine learning (ML). These techniques are applied to the studies of protein interactions, drug binding affinities, and genetic mutations [46]. Resources such as MechPPI utilize protein–protein interaction networks in order to prioritize resistance candidates. The binding of molecules is very sensitive to hydrophobic region, hydrophilic interaction, and hydrogen bonds according to the prediction effects of MechPPI, the mutation analysis [39]. PSnpBind-ML uses large datasets for accurately predicting changes in binding affinity upon mutations and predicts the influence of single-nucleotide polymorphisms (SNPs) on binding affinities of ligands [47]. Additionally, structure-based modeling and free energy perturbation (FEP) simulations can be used to computationally predict drug binding to mutated targets, providing a quick in silico screen to test new antibiotic leads against resistant strains. These tools are especially useful for the detection of resistance mechanisms before they are clinically prevalent and can inform pre-emptive treatment adaptations. But computational predictions need experimental validation, and their accuracy depends on the quality and the coverage of the training data. Computer or in silico modeling offers invaluable tools, as an adjunct to laboratory-based research, to predict and detect resistance as it emerges and to intervene proactively by predicting resistance mutations and offering strategies for manipulating drugs.

The drug-resistance-associated genetic changes fulfil both functions—they are potential leukemia treatments, targeted at resistant elements, and are individualized drugs. Inspection of these drug-resistant interactions provides a foundation for therapeutic development and drug-action optimization (Figure 2). The binding changes of drugs have significant implications for both patient and disease therapy. Changes in plasma protein binding alter unbound drug levels, impacting both efficacy and toxicity. Research shows that over time, the benefits of Disease-Modifying Drugs (DMDs) used in patients with rheumatoid arthritis (RA) and multiple sclerosis (MS) gradually disappear, thus delaying long-term treatment effectiveness [48]. RA and MS treatments losing efficacy serves as a conceptual model to understand how repeated drug exposure and molecular adaptation can lead to treatment failure, underscoring the urgency of addressing AMR through both preventive and adaptive therapeutic design. Some medications develop covalent bonds with specific proteins, which creates toxic effects that could trigger targeted organ damage and uncertain medical results [49]. The need for ongoing drug effect evaluations is clearly important because early therapeutic reactions do not ensure safe or effective management in the long term [50]. To summarize, interference in the interaction between drugs and proteins, either by modifying the target or by altering binding kinetics, directly affects antimicrobial efficacy and contributes to the development of resistance.

### 2.2. Challenges Posed by AMR to Conventional Therapies

The effectiveness of traditional therapeutics, such as those against bacterial infections, is still weakening as a result of AMR. Since the level of resistance rises among both community-acquired and nosocomial pathogens, physicians are experiencing the restriction in drug availability, decreased success of therapies, and an increase in healthcare costs. These problems are compounded by a flat antibiotic development pipeline and growing worry over both the safety profile and the potential lack of specificity of new genomic and RNA-based therapeutics. In this section, the principal clinical and pharmaceutical problems presented by AMR are described and classified on the basis of their thematic relevance to provide a clear structure.

The impact of AMR on clinical outcomes is catastrophic. Infections that were once easy to treat—such as urinary tract infections, pneumonia, or infections after surgery—are proving increasingly resistant to antibiotics at the first level of defense. *Klebsiella pneumoniae*, *Acinetobacter baumannii*, and methicillin-resistant *Staphylococcus aureus* (MRSA) are some of the multidrug-resistant (MDR) organisms that are common now both in hospital and community settings and often lack suitable, non-toxic, or less ineffective alternative therapies. Treatment based on resistance patterns may lead to delays in treatment, long hospital stays, exacerbation of the disease, and an increase in mortality and morbidity. Furthermore, the absence of appropriate oral antibiotics for outpatient treatment of resistant infections poses an additional infection control challenge to inpatient services.

Although new antibiotics are urgently needed, drug development is limited by high costs, low returns, and strict regulations. These challenges have discouraged investment in antimicrobial innovation [51]. According to WHO (2024) statistics from the WHO AMR Pipeline Tracker (2025), fewer than 12 novel systemic antibiotics are in advanced clinical trials—a stark contrast to over 30 candidates in the 1990s. The decline of antibiotics research funding created an unacceptably small pipeline of newly developed drugs [52]. Developments in antibiotics research and innovation require immediate incentives to operate effectively. Current research seeks new therapeutic methods because antibiotics are demonstrating a decline in efficacy in treatments. Many of the few newly authorized antibiotics target the same resistance mechanisms and therefore offer only limited innovation. In addition, regulatory hurdles and the need for stewardship measures hinder the widespread use of new agents, further impeding their commercial development. Taken together, these factors create a critical innovation gap in dealing with emerging AMR threats.

New treatment strategies based on RNA interference (RNAi), CRISPR-Cas, and antisense oligonucleotides have the potential to be excellent alternatives to antibacterial treatment. By targeting essential genes and pathways, they block bacterial replication, virulence, or resistance [51,53]. Nevertheless, these methods also bring about new challenges. Off-target effects—including the accidental silencing of genes (*siRNAs* and *miRNAs*), immune stimulation, or interactions with the microbiome of the host—are associated with significant safety issues [54]. Off-target effects are unintended results or activities that take place when a drug or genome editing technique such as CRISPR-Cas9 engages with molecules or sequences of DNA other than its target [53]. For instance, if we attempt to silence a bacterial gene, we run the risk of inducing an accessory effect which would lead to the inhibition of non-target bacteria, including the ones with positive properties in the microbiota. The genome editing domain faces numerous difficulties associated with unwanted mutations in its targets. Technologies used to modify genomes unpredictably adjust similar genomic sequences, which creates dangers for destructive genetic modifications that strengthen resistance competencies [55]. Accurate off-target screening methods developed under ABSOLVE-seq emphasize the necessity of genome editing precision to stop drug-resistant mutations from occurring in therapeutic sequences. Osteosarcoma treatment resistance against chemotherapy develops through drug breakdown mechanisms alongside DNA repair system enhancement, and resistance pathways are worsened by treatment-related side effects [56]. The disease-fighting potential of hybridization-dependent relations between treatment molecules results in unintended gene silencing effects, which enables cancer cell resistance development. Hybridization-dependent relationships refer to how the process of hybridization, in which two complementary strands of nucleic acid join together, can influence various biological interactions and processes. The delivery of these therapies also remains a major hurdle, particularly when targeting intracellular or biofilm-embedded pathogens. Although RNA- and CRISPR-based interventions could complement future antimicrobial strategies, they currently face significant implementation and safety limitations.

Since there are not many new drugs, it is important to improve the use of the available antibiotics. Antimicrobial stewardship programs (ASPs) try to balance providing appropriate treatment by reducing the selection pressure for resistance. These include the guideline-based prescribing and antimicrobial stewardship programs, treating cases with de-escalation guided by culture results and time to limit the patients’ use of the antibiotics [52]. The infection prevention and control (IPC) measures—hand hygiene, environmental hygiene, and vaccination—act synergistically to prevent the spread of resistant organisms [51]. Meanwhile, enhanced global surveillance and rapid diagnostic methods are crucial to facilitating real-time decision-making and outbreak response. Indeed, effective containment of AMR will demand a coordinated approach which combines drug development, therapeutic innovation, stewardship, and public health infrastructure.

In conclusion, to address AMR, a “whole-system” approach is required. This encompasses drug discovery, surveillance, stewardship, and public education and spans such advanced technologies as nanomedicine and personalized therapy.

## 3. Role of Targeted Drug Delivery in AMR

### 3.1. Benefits of Targeted Drug Delivery

Targeted drug delivery aims to enhance the concentration of medication in specific tissues while minimizing its presence in other areas. This method can enhance the efficacy of the therapeutics and minimize the off-target effect [57]. Fighting AMR has been a struggle as pathogens quickly adapt and evolve different mechanisms to avoid antibiotics. One of the most important resistance mechanisms is the complex multilayered envelopes, which protect the microbes and constitute a significant filter for the majority of anti-infective agents. In addition, the mammalian cell membrane is another barrier for antibacterial agents against intracellular pathogens. To overcome these biological barriers, researchers have created drug delivery systems that enable treatments to penetrate cell walls—commonly referred to as “Trojan horse” approaches [57]. Furthermore, targeted drug delivery ensures the precise transport of therapeutic agents to the infection site, as demonstrated in several preclinical and clinical studies. So, the inherent benefit of this method has been the reduction in dose and side effects of the drug [58]. In many therapeutic medical fields, TDDSs proved their effectiveness in treatment improvement. For instance, improved delivery of chemotherapeutic drug delivery to human tumor tissue seems to be attainable [59]. At present, various strategies, such as combinatorial therapy, chemical modification of antibiotics, photothermal agents, antimicrobial peptides, cationic polymers, and nanoparticles, have been reported to be auxiliary for combating antibiotic resistance [60].

**Figure 3:**
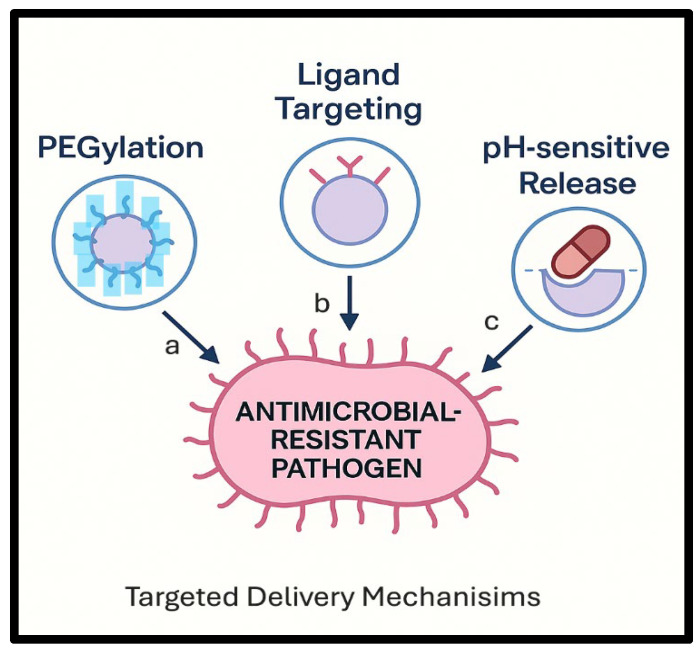
Enhancing drug concentration at the site of action is achieved through several mechanisms (image created with Canva software (Version 4.0, Canva Pty Ltd., Sydney, Australia).

Low concentrations of intracellular drug, the development of MDR, drug efflux by efflux pumps, and enzymatic degradation are significant drawbacks of traditional therapy [61]. However, when the drug is targeted to specific sites, it reduces exposure to healthy tissues, which minimizes the risk of systemic side outcomes commonly noticed with conventional drug delivery procedures [62]. Enhancing drug concentration at the site of action is achieved through several mechanisms (Figure 3): Firstly, increased drug accumulation at target site by nanocarriers (liposomes, nanoparticles, micelles) [63] and ligand–receptor targeting. Nanocarriers guard drugs from degradation and ensure their release at the specific site. Drug carriers are modified with ligands (such as monoclonal antibodies for cancer cells) that bind selectively to receptors on sick cells [64]. Secondly, in reduced systemic clearance by PEGylation, a polyethylene glycol (PEG) coating prevents recognition by the immune system, which leads to a prolonged circulation time [64]. Controlled release formulations are another way to reduce systemic clearance. So, slow-release polymers minimize drug breakdown before reaching the target site. Finally, improved cellular uptake is achieved by PH-sensitive delivery and endocytosis targeting [65]. Zhao et al. developed antimicrobial peptide defensin-loaded mesoporous silica nanoparticles (MSNs) aimed at targeting the intestine [66]. Defensin is prone to degradation in the stomach, and to ensure effective targeting of the intestine, they coated the surface of the MSNs with succinylated casein, which can be broken down by intestinal protease. The casein coating reduced the release of defensin in an acidic environment, whereas a controlled release pattern was observed in the presence of trypsin. Multidrug-resistant *E. coli* was introduced through oral gavage to induce intestinal infection. The nanoparticles were administered orally each day for five days, and the casein-coated nanomedicine significantly reduced bacterial colonization compared to free ciprofloxacin, which served as the positive control. Also, the level of the proinflammatory mediator TNF-α in the intestine decreased by 1.5- and 2.2-fold after the application of the casein-coated nanoparticles, in comparison to non-coated MSNs and free peptide, respectively.

Advancements in drug delivery technologies are enabling more effective and less toxic treatment regimens. TDDSs formulate and store drug molecules in suitable forms like solutions or tablets for administration. They enhance drug distribution to their precise site of targeting in the body, resulting in improved therapeutic outcome and decreased off-target deposition in the body [59]. Certain approaches of targeted drug delivery systems have resulted in significantly reduced toxicity and systemic toxicity of antimicrobials. For instance, targeted therapy nanocarrier-mediated drug delivery is also in high demand in that it allows drugs to be specifically delivered to the sites of tumors or infection, where the action occurs with fewer side effects on normal cells and tissues. Enhanced bioavailability: Nanocarriers can change the distribution and pharmacokinetics of drugs in tissues, increasing their uptake by the cells and bioavailability. However, this may result lower doses of drugs used, resulting in a reduced total systemic exposure. Extended circulation: Nanocarriers can enhance the stability of drugs and prolong their presence in the bloodstream, thus reducing the need for frequent administration and the overall dosage required. Various delivery vehicles such as liposomes are also effective in preventing drug degradation; thus, a lower dosage is required that also reduces side effects. Activation of the prodrug only at infection sites may minimize systemic off-target side effects and allows for high-level local concentration [67]. Antimicrobial peptides (AMPs), a new type of antibiotic, present relatively effective broad-spectrum antimicrobial activity against a number of MDR pathogens. The obtained dimeric peptide was demonstrated to be very potent with respect to WHO prioritizing MDR *A. baumannii* as representative Gram-negative bacteria with no cytotoxicity. Time kill kinetics revealed that the dimeric peptide was bactericidal and it killed over 50% of preformed bacterial biofilm. This information serves as pharmacodynamic information for more extensive clinical pharmacokinetic studies and potential therapeutic development [68]. Biofilms and intracellular infections are two complex and distinct challenges that remain a serious concern in clinical services. The high resistance of biofilms to antibiotic therapies seems to be a major obstacle in this field [69]. Biofilm displays a tough structure and significant resistance that obstructs the entry of antimicrobial medications [60]. Compared to planktonic cells, biofilm cells exhibit at least hundreds of times greater resistance to antibacterial agents (up to a 1000-fold increase) [70].

**Figure 4:**
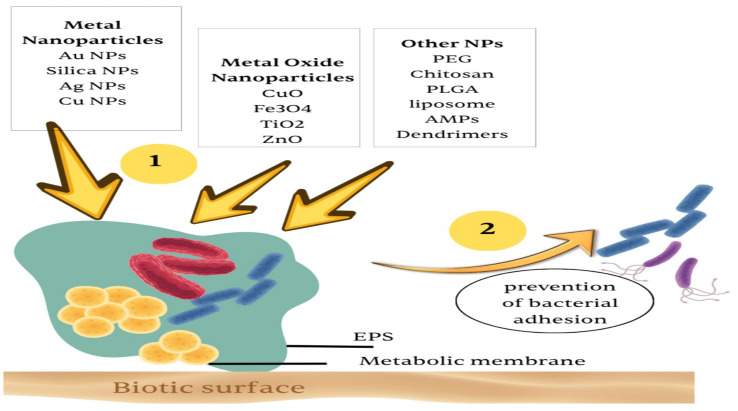
Types of nanomaterial-based treatment of bacterial biofilm (image created with Canva).

Biofilm-dispersing enzymes break down the extracellular polymeric matrix that envelops bacterial biofilms, disperse the microbial community, and make them more vulnerable to immune cells and drugs [71]. Biofilm bacteria are vulnerable to antimicrobials when cultivated in a standard laboratory suspension culture. Several aspects of the biofilm development process have been investigated as potential targets for innovative drug delivery systems [72]. Targeted drug delivery systems offer promising and valuable solutions for overcoming biofilm barriers and treating intracellular infections. These systems aim to deliver drugs directly to the target site to improve efficacy [67]. Certain nanoparticles possess the ability to disrupt biofilms, allowing easier access for penetration and the eventual elimination of bacteria. Enhancing the surface characteristics of nanoparticles facilitates their substantial absorption into host cells, leading to effective bacterial removal. The USFDA has already approved 51 nanomedicines, which include nano-based formulations designed for antibacterial purposes [73]. The basic types of nanocarriers include molecular complexes (like protein and cyclodextrin nanocomplexes), polymer-based nanocapsules (including dendrimers and core–shell structures), inorganic nanocarriers (such as metal nanoparticles), and lipid-based nanovesicles (such as liposomes and solid lipid nanoparticles) (Figure 4). Among the various nanomaterials, nanoparticles have garnered significant interest [74]. Acting as carriers, nanoparticles can improve drug solubility and stability while also enhancing drug biocompatibility at the targeted site [75].

One study explored a potential therapeutic approach for lung infections by optimizing cationic nanosized liposomal formulations loaded with an antibiotic and an antibiofilm peptide to evaluate their efficacy against MDR strains of *P. aeruginosa*. It concluded that the biofilm formation was significantly reduced (*p* < 0.05) at concentrations of ≥4 μg/mL and ≤32 μg/mL when loading tobramycin into liposomes, with or without the anti-biofilm peptide, compared to the free tobramycin antibiotic [76]. NPs have different strategies for accessing and interrupting bacterial biofilms (Figure 4). These strategies include targeting biofilms with preventing initial attachment, interfering with biofilm formation, disrupting the EPS (extracellular polymeric substance) matrix, disrupting mature biofilms, and decreasing biofilm regeneration [77].

### 3.2. Comparison with Conventional Delivery Methods

Developing a valid method to address AMR should address the drug design, a delivery route, and in addition the delivery system for targeting. In humans, antibiotics are usually delivered orally, intravenously (IV), intramuscularly (IM), or topically or are inhaled [78]. To travel from the gut to the bloodstream, oral antibiotics must traverse the walls of the gut. Topical antibiotics must be able to penetrate the stratum corneum to be absorbed through the skin. Antibiotics used to treat pulmonary infections must traverse the mucosal layers. When bacteria exist within cells, antibiotics encounter the additional challenge of crossing the host cell membrane and accessing the sub-cellular area where the bacteria are located. For effective treatment, it is necessary for the antibiotic to achieve a sufficient concentration at the infection site for a designated period [79].

Though widely used, most of the standard drug delivery systems suffer from several limitations like non-specific distribution, poor bioavailability, poor tissue penetration, systemic toxicity, and adverse effects on the microbiome [79,80,81]. A summary of the comparative shortfalls of conventional and targeted delivery systems is presented in Table 1 by comparing these strategies based on the critical clinical parameters. This contrast illustrates the fundamental advantage of targeted systems: their ability to reduce off-target effects responsible for AMR generation and toxic side effects, as well as to enhance treatment efficiency at the site of infection.

Classic chemotherapeutics, which have revolutionized public health, suffer, however, from limitations that factor into AMR. The prevalence of infectious diseases has decreased since the 1920s following the discovery of antibiotics and their commercialization in the 1940s [82]. A number of dramatic medical advances of the 20th century were associated with the discovery and manufacture of antibiotics [83]. Non-specific antibiotics that indiscriminately inhibit bacterial processes like nucleic acid synthesis (sulfonamides and quinolones), protein synthesis (tetracyclines and aminoglycosides), or cell wall synthesis (beta-lactams and vancomycin) promote AMR due to their non-specificity and delivery failure [84]. Their nonselective effect disrupts the balance of microbial ecosystems and favors resistance, reducing, in particular, the administration’s ability to distinguish between pathogens and commensal bacteria [85,86]. The main examples [84,87] are beta-lactams (e.g., penicillin), with an effect on the synthesis of the cell wall, which due to poor tissue penetration are given in high systemic doses, increasing risk of hepatotoxicity and hypersensitivity reaction; aminoglycosides (for example, gentamycin), with generalized renal retention that leads to nephrotoxicity and can occur in individuals predisposed [88]; fluoroquinolones (ciprofloxacin, for example), which have broad-spectrum action with gut microbiota destruction that knocks out colonization resistance and allows such bacteria as *Clostridium difficile* to prosper [89,90]. Years of misuse and overuse of antibiotics have further compounded this problem, leading to AMR both in Australia and internationally [89]. These challenges highlight the immense necessity for new generations of drug delivery platforms, with optimized targeting, to reduce off-target and resistance effects.

Broad-spectrum antibiotics have an extensive negative effect on the gut microbiome, with effects even occurring in appropriate clinical settings. Although every class of antibiotics disrupts microbial communities, some inflict disproportionately severe damage. For instance, oral antibiotics frequently perturb the gut microbiota due to the inhibition of commensal bacteria and the reduction in colonization resistance [91,92]. Ciprofloxacin, a broad-spectrum fluoroquinolone, elicits much more profound perturbations in the gut microbial community structure than narrower-spectrum antibiotics such as amoxicillin [89,90]. This is also the case for broad-spectrum antibiotics, and in the process, they also kill the non-threatening bacteria that would help keep the harmful bacteria in check. Its effects are not only persistent during administration of antibiotics but also for a period of time afterwards. This shows that a brief exposure to broad-spectrum antibiotics can decrease the relative abundance of gut microbiota relative to pre-antibiotics for up to two years [14,85]. Macrolides are not considered as HRBSA but cause persistent modulations of the gut microbiome [85,93]. In a worst-case scenario, antibiotic-driven alterations to the microbiome could be permanent and have long-term health consequences. Abnormalization of microbiome function influences a variety of important roles of the microbiome, e.g., vitamin synthesis, substratum supply, and pathogenicity protection, leading to susceptibility to diverse infectious diseases in human beings [14].

A diverse, healthy gut microbiota is essential for preventing AMR via colonization resistance, a process that limits pathogen overgrowth [94,95]. The breakdown of this barrier resulting from dysbiosis, or perturbation of the microbiota, is implicated in multiple chronic diseases. This is evidenced by the strong association with metabolic diseases, including type 2 diabetes, obesity, and nonalcoholic fatty liver disease, or inflammatory diseases such as inflammatory bowel disease (IBD) or repeated *Clostridium difficile* infections, with taxonomic alterations in gut communities [96,97,98]. Importantly, dysbiosis also has a deleterious effect on colonization resistance, giving the host increased susceptibility to antibiotic-resistant pathogens such as VRE (Vancomycin-resistant enterococcus) and *C. difficile* [94,99]. In addition, these disturbances cause metabolic alterations and decrease the diversity of the microbiota and the emergence of resistance in pathogens [96]. For example, antibiotic-induced dysbiosis could lead to the development of antibiotic-associated diarrhea and could create an environment that promotes the survival of *C. difficile* [100]. Apart from AMR hazards, chronic dysbiosis has been linked to autoimmune and atopic diseases, further mirroring its systemic health implications [101]. Therefore, maintaining the microbiome is essential for health and for preventing antibiotic-resistant infections.

Conventional management of antibiotics is generally ineffective in achieving a sufficient concentration at the site of intracellular infection because of poor membrane permeability, stability, and bioavailability [102]. It induces subtherapeutic levels at the site of infection, demanding higher or frequent dosing, which may be the main cause of toxicity and resistance [61,82]. Drug pharmacokinetics following routine antibiotic delivery are commonly characterized by fluctuations that too often translate into bacteriostatic levels that are too low and/or bactericidal levels that are too high. For example, some (fluoro)quinolones exhibit a propensity for selective accumulation in certain tissues, including cartilage, with the associated faculty for locoregional toxicity [103]. AST could be used to treat a variety of infections, including ocular and central nervous system infections, as application of many broad-spectrum antibiotics was limited due to the physiological barriers such as the blood–ocular barrier and blood–brain barrier [104,105]. This limitation of drug delivery can lead to unsuccessful treatment and ongoing barriers posed by resistant pathogens.

Despite their effectiveness in eliminating bacteria, antibiotics can produce undesirable responses in humans, often due to ineffective medication regimens. Resistance mechanisms are mostly determined by pharmacokinetics or pharmacodynamics models, resistome analyses, and antibiotic toxicity [106]. Recommendations have been made to restrict the use of fluoroquinolones to fewer severe diseases due to their serious side effects, despite their effectiveness as broad-spectrum antibiotics [107,108]. Antibiotics, such as aminoglycosides (e.g., gentamicin), can accumulate in the kidney’s proximal tubules and cochlear cells, potentially leading to nephrotoxicity and ototoxicity [109]. Also, one potential side effect of spectinomycin and other aminoglycosides is respiratory paralysis. This condition is generally reversible with calcium gluconate [109]. Antibiotics containing fluoroquinolones may cause peripheral neuropathy, tendinopathy, aortic dissections, or aneurysms and can negatively affect the CNS [103,108,110]. Therefore, if alternative treatments are available, it is advisable not to use fluoroquinolones as a first-line treatment for common infections, such as acute sinusitis, bronchitis, and urinary tract infections [111]. Antibiotics are the commonest cause of severe immune-mediated drug reactions considered to be off-target, including anaphylaxis and severe reactions in selective organs and skin. More precisely, a label of penicillin allergy is associated with an increased use of broad-spectrum and non-β-lactam antibiotics, which in turn leads to increased adverse effects and further promotes antibiotic resistance [112].

The rise in AMR has driven the advancement of precise medicine and targeted drug delivery technology. These approaches selectively act on molecular or cellular markers that can enhance drug efficacy and decrease systemic exposure in the body [113]. Antibiotics targeted to the site of infection limit the concentration of most drugs to the local infection without causing side effects and resistance in commensal microbes [114,115]. The fundamental targeting ability is accomplished through several advanced mechanisms, which include surface functionalization with ligands for receptor-mediated uptake, antibody-mediated recognition of pathogen-specific antigens, and responsiveness to distinctive pathological stimuli like low pH or specific enzymes [79,116,117]. To this end, advanced delivery systems may be engineered to control release of antibiotics, circumventing the bacterial resistance mechanisms, as well as the potential for resistance emergence [8]. These nanoparticles possess better solubility, stability, permeability, and bioavailability, and less toxicity, than the formulations prepared by the traditional methods [118,119,120]. Nanoparticles enter into the host cell membrane via endocytic and phagocytic routes to target the intracellular pathogens, upon which the classic antibiotics are not able to act [102]. The potential of functionalized nanoparticles with targeting moieties such as peptides or antibodies with the ability to cross the blood–brain barrier has been shown in various works, and therefore compositions could also potentially be used for CNS infections [121]. Additional systems are pH-responsive nanoparticles that release antibiotics in acidic environments like bacterial biofilms for drug exposure and penetration [122,123].

The liposome-mediated delivery of drugs is considered very promising. Liposomal antibiotics are targeted drugs, with fewer side effects and with protection against enzymatic breakdown. Liposomes permeate biofilms of bacteria, and that also enhances their efficacy in resistant bacterial strains, including methicillin-resistant *Staphylococcus aureus* [124]. Both *Pseudomonas aeruginosa* and *Escherichia coli* biofilms were challenged by liposomes with success. Moreover, liposomes can be engineered with the addition of specific ligands or antibodies in order to more precisely target a specific part of the body [124].

Another alternative is to employ antibody–antibiotic conjugates. Chimeric constructs of antibodies and antibiotics connect themselves, which concentrates their effect on bacterial antigens, which in turn limits systemic toxicity and associated side effects [116,125]. The antibody component targets the pathogen by attaching to certain surface markers, while the antibiotic payload is delivered directly to the bacterium [116]. Besides the aspects cited above, siderophores, cell penetrating peptides, and bacteriophages are currently thought to be involved in assisting antibiotics penetration through different body barriers [126]. These developments have many implementations, from countering pools of infectious bacteria to experimental treatments, and at a systems level, all contribute to overcoming the challenges of infections and resistance, which include AB stewardship, infection control, surveillance, and candidate drugs [127].

**Table 1:** Comparison of targeted drug delivery with conventional delivery methods.

Factors	Conventional Delivery Methods	Limitations of Non-Targeted Antibiotics	Targeted Drug Delivery
**Mechanism of Action**	Broad-spectrum actions target multiple bacterial species [89,90].	Random targeting impacts beneficial microbiota and enables AMR [14].	Targeting antimicrobial agents directly at the site of infection or the pathogen reduces their contact with non-target regions [80].
**Specificity**	Non-targeted methods impact both pathogenic and non-pathogenic bacteria [10].	Inadequate selectivity disturbs beneficial microbiota and decreases colonization resistance [14,116].	Specifically, it focuses on areas of infection or harmful microorganisms [79].
**Efficacy Against Biofilms**	Efficacy is limited due to insufficient penetration and activity in biofilms [21].	Conventional treatments are ineffective against biofilm-associated infections [21].	It is effective against biofilms [122].
**Accumulation and Penetration**	Penetration into cells is poor [87].	Low-penetration efficiency results in a high dose being required [87].	It targets intracellular pathogens by facilitating drug entry into cells [102].
**Side Effects and Toxicity**	Frequent systemic side effects result from non-specific actions [14].	Excessive dosage and fluctuating medication levels above the therapeutic range provide a toxicity risk [82].	Drugs can be encapsulated in carriers like liposomes and nanoparticles to preserve healthy tissues, increase localized dosages at infection sites, and decrease systemic toxicity overall [116].
**Bioavailability**	There is low bioavailability and quick clearance [102].	Higher dosages are necessary due to inefficiencies at infection sites caused by limited medication uptake through biological membranes [102].	Nanocarriers and delivery methods are beneficial since they can combat early degradation and extend the half-life of antibiotics, improving medication stability and bioavailability [118].
**Emergence of Antibiotic Resistance**	Overuse and misuse make resistance possible [128].	The overall emergence of antibiotic-resistant pathogens is increasing, with a special threat coming from those that are associated with the formation of biofilms [22].	Targeted systems produce greater localized levels of antibiotics at the site of infection, effectively addressing drug resistance mechanisms [114].
**Impact on the Economy and Healthcare**	Costs associated with long-term therapy and equipment replacement are considerable [17].	Long-term infections are a cause of increased duration in hospitals and increased costs to the patients [17].	Targeted drug delivery systems pose the potential to positively affect the economy and the healthcare industry by improving the precision of the treatments [127].

## 4. Innovative Strategies in Targeted Drug Delivery

### 4.1. Nanoparticle-Based Systems

Nanoparticle-based approaches have been widely explored in the last decade to address AMR [124,129,130]. These particles, usually from 1 to 100 nm in size and up to 500 nm in some cases, have powerful capabilities despite their minuscule length [131,132,133]. Their range of structures also mean they are very good at targeting drugs to infection sites, maximizing the treatment of infections [132]. Within these approaches, a number of nanoparticles, such as liposomes, polymeric nanoparticles, and solid lipid nanoparticles, have provided new alternatives.

**Liposomes** are spherical lipid bilayer vesicles (similar to cell membranes) able to encapsulate hydrophilic and hydrophobic drugs, which is a characteristic that differentiates them from other microparticle systems [124]. The physical character of liposomes is an important factor in stability and biological activity in vivo and in vitro. Recently, a few liposomal products have been approved by the FDA for infections [124]. Encapsulation of antibiotics into liposomes permits direct targeting of the drug to the site of the infection, which can enhance the efficiency of therapy. Modifying these particles to be responsive to biological microenvironments increases the half-life of antibiotics in circulation [124,134,135]. For instance, colistin-enclosed liposomes have been found to be effective as colistin solution for treatment of infections [136]. One in vivo study found that mice infected with *Pseudomonas aeruginosa*, in which colistin-loaded liposomes were used as the formulation, showed a superior survival time compared to those treated with blank liposomes and colistin solution [137]. Additionally, colistin liposomes decrease the systemic toxicity by preventing many parts of the body from being affected [137].

**Solid lipid nanoparticles (SLNPs),** as the name suggests, comprise a solid lipid matrix with entrapped drugs that is stabilized by surfactants [132,138,139]. These nanoparticles entered the mainstream in recent years following their use in encapsulating mRNA for Moderna and Pfizer vaccines against COVID-19 [138]. Among a list of advantages, SLNPs have high drug-loading capacity, enhanced stability, and they can be used at large scale [140]. Different studies reported that SLNPs loaded with AMPs increased the curing of infections when given orally or topically. For example, Lactine 3147-loaded SLNPs resulted in increased antimicrobial activity against *Listeria monocytogenes* and protected the peptide from degradation by alpha-chymotrypsin [141]. Similarly, SLNP-loaded polymyxin B has been found to exhibit bactericidal activity against six distinct AMR strains of *P. aeruginosa*. Furthermore, the LL-37- and Serpin A1-containing preparations have facilitated the healing of chronic wounds [142,143].

**Polymer nanoparticles**, composed of natural or synthetic polymers held together in covalent bonds, are further classified according to composition, i.e., polyesters, polyanhydrides, and polysaccharides [144,145,146]. All of these subtypes have distinct advantages for use in specific applications. For example, polyesteric nanoparticles are famed for biocompatibility in biochemical conditions. Polyanhydride nanoparticles are highly prized for their superior performance as drug delivery systems due to their ability to facilitate the rapid release of AMPs. Lastly, polysaccharide nanoparticles are considered the second-best option for delivering AMPs [132,145].

**Nanoparticles (NPs)** interact with bacteria in various manners to facilitate their removal. The topology of the cell walls of bacteria is a significant consideration in this interaction. For example, Gram-negative bacteria possess an advanced wall structure comprising lipopolysaccharides (LPSs) with a negatively charged surface area that binds NPs, while Gram-positive bacteria possess a penetrable cell wall comprising peptidoglycan and teichoic acids that are susceptible to NP penetration [147,148,149]. Studies indicate that NPs target Gram-positive bacteria more effectively because of their penetrable cell walls, and Gram-negative bacteria resist NP adhesion due to their LPS layer [149]. When they come into contact with bacterial cells—by electrostatic attraction, van der Waals forces, or hydrophobic interactions—NPs enter the cell membrane, targeting key cellular components such as DNA, enzymes, and metabolic processes, inducing oxidative stress, altered membrane permeability, and ultimately cell death [150,151]. Moreover, NPs exert inhibitory effects on biofilms, which are protective layers that promote bacterial resistance, by aggregating with extracellular polymeric substances (EPSs) and by altering bacterial adhesion and metabolism [149,152,153,154]. Based on these multi-mode interactions, NPs present a new strategy for the prevention of bacterial infection, especially in the post-biotics resistant era.

### 4.2. Stimuli-Responsive Systems

Stimuli-responsive systems are also ideally designed to release their drugs as a function of changes either occurring in the environmental conditions or in response to trigger events from inside and outside the biological barrier, i.e., pH [155], temperature [156], and enzymes [75] (Table 2). Such pH-responsive systems use the differences in pH of tumors compared to normal tissues, such as a pH of less than 5.7 due to anaerobic glycolysis with fermentation of glucose to lactic acid within tumor tissue. For instance, inflammatory islands, tumor microenvironments, and biofilms tend to have lower pH compared to healthy tissue [81,157]. pH-responsive carriers are those which remain stable at physiological pH but release their cargo when placed in a lower pH environment [158]. These nanoparticles either disintegrate or degrade in response to acidic environments that in turn lead to release of the maximum amount of drug at site of action [158]. When applying the bacteriophage, because biofilms can acidify the medium and decrease the pH, the biofilm lowering the pH in the mouth related to gingivitis and caries can be broken [158]. For instance, the development of a pH-responsive drug delivery carrier for *A. baumannii* infection control was reported in one of these recent studies and exemplifies innovative approaches and their likely contribution to address urgent health needs [159].

Temperature-sensitive systems, such as pH-sensitive devices, are designed to release drugs on the basis of temperature changes [156,160]. This may be locally due to pathological states such as inflammation whenever local temperatures rise or can be externally via applying heat over a specific site in the body in order to trigger drug delivery [156].

Enzyme-sensitive systems are driven by the presence of certain enzymes that are often overexpressed inside infected cells or excreted by particular bacteria. Lipases, for instance, tend to be excreted by Gram-negative and Gram-positive bacteria [49,75]. In environments with high enzyme levels, lipase-sensitive drug delivery systems can be effectively employed to deliver therapeutic agents [49,75].

### 4.3. Bacteriophage-Based Delivery

The emergence of MDR bacteria worldwide poses a major threat to public health. This problem has renewed interest in bacteriophages (phages) as antimicrobials, either as a sole therapy or in combination with antibiotics. Phages offer unique properties that help to combat bacteria and antibiotic resistance, either through synergy with antibiotics or as new delivery systems. It has been suggested that combination treatment with both, rather than with one alone, results in an increased bactericidal effect [161]. The phage and combinatorial antibiotic therapy of various Gram-positive and -negative bacteria has been systematically reviewed. It was demonstrated that the combination of phages and daptomycin was able to greatly increase the elimination of *E. faecium* strains, including those with daptomycin resistance. There was also a significant enhancement of bacteria removal associated with decreases in the number of phage- and antibiotic-resistant mutants. Moreover, in *P. aeruginosa* biofilm models, the combined treatment of phages with antibiotics was capable of markedly activating the biofilm destruction and eradication of bacteria, suggesting the capacity of phages in treatment of persistent biofilm-related infections [161]. Bacterial filaments may develop when phages and antibiotics are conjugated, resulting in enhanced phage multiplication, a larger burst, and an increased sensitivity of bacteria to phage lysis. Additionally, the degradation of bacterial polysaccharides by phage-derived enzymes facilitates the entry of antibiotics into biofilms. This synergistic effect enables the use of lower antibiotic dosages, which reduces the selection pressure that would cause resistance to develop [161].

The use of phages as carriers to move compounds that target bacteria from one location to another is a novel therapeutic strategy. Wang et al. highlighted the possibility of chemically and genetically altering phages, such as the filamentous phage M13, to enable them to transfer genes, antibiotics, nanoparticles, or a mixture of these to a particular bacterial target [77]. Phages could potentially be attached to antibiotic complexes, liposomes, or nanoparticles. This would allow for more specific targeting of bacteria that have developed resistance compared to these compounds alone. Researchers are also investigating the possibility of establishing treatments using complexes comprising a phage and a silver nanoparticle, which have been shown to be particularly effective at eliminating bacteria. The modulation of the gut microbial profile and reprogramming of cancer-related dysbiosis are some of the potential applications of these complexes [77]. They can also re-sensitize resistant bacteria to antibiotics. There is evidence that colistin-resistant strains of *Acinetobacter baumannii* can become susceptible following exposure to a phage preparation, demonstrating a reversal of resistance mechanism, which could be exploited in clinical practice.

Engineered phages are also employed to deliver endolysins as well as genetic material that could influence the activity of the bacterium. Lin et al. discussed lysins and CRISPR-based tools as novel therapeutic agents. They referred to how these constructs are capable of rupturing cell walls or blocking resistance genes in bacteria. This broadens the application of phages, from the infection of bacteria to the specific gene expression [162].

In short, bacteriophages are an important tool in the fight against antibiotic resistance since they can either directly kill bacteria or function as carriers for antimicrobial drugs, increasing their potency. As a result of the ongoing decline in the effectiveness of traditional antibiotics, the specificity and flexibility of phages make them ideal candidates for use in new antimicrobial therapies. As such further research in phage engineering safety profiling and clinical standards, particularly in the theme of utilizing phages as anti-microbe delivery systems, is highly anticipated.

### 4.4. Antibody–Drug Conjugates (ADCs)

ADCs are a novel technology in which an antibody is used to direct a chemotherapeutic specifically at pathogenic bacteria, therefore leaving the host microbiota intact [163]. ADCs are a result of positive outcomes of serum therapy from discoveries and developments in the production and evolution of antibodies [164]. The choice of the antibody subclass is important for achieving optimal efficacy [165]. Bispecific antibodies, possessing two different antigenic specificities, exhibit huge advantages in targeting pathogens and improving the quality of diagnostic testing [166], offering certain advantages over conventional antibiotics, such as specific action on pathogenic microorganisms and reduced potential emergence of resistance [164,167]. They are distinguished mostly by their high antibacterial activity with many monoclonal antibodies (mAbs); these chemical therapeutic agents possess the specificity of mAbs and antibacterial activity. Furthermore, in vitro and in vivo research has shown that ADCs have antibacterial activity against *P. aeruginosa* and other Gram-negative MDR bacteria, without host cell toxicity [163,168]. Specific antibodies have also proven effective for controlling infection by *Clostridium difficile* [169]. Furthermore, ADCs have been investigated for their use against viral infections and as a biodefense medication against prospective biological weapons [170,171].

**Figure 5:**
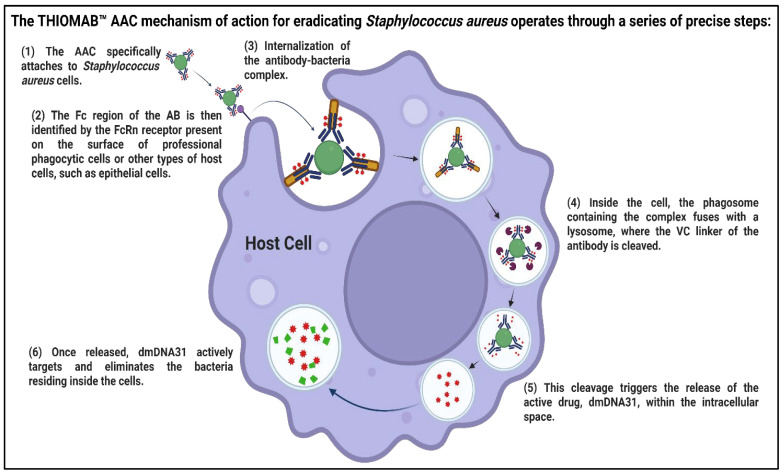
The THIOMAB™ AAC mechanism of action for eliminating *Staphylococcus aureus*. Mechanism of action of the THIOMAB™ AAC against *Staphylococcus aureus*: Initially, the AAC binds specifically to *S. aureus*, enabling specific targeting. The Fc portion of the monoclonal antibody is then identified by FcRn found on the surface of phagocytes or other host cells, such as epithelial cells. Subsequently, the AAC–bacteria complex is then internalized by the host cell. Within the cell, the phagosome containing the complex merges with a lysosome, leading to the cleavage of the VC linker. Then, the active compound, dmDNA31, is released into the intracellular environment. DmDNA31 targets and eliminates intracellular bacteria (created with BioRender.com).

ADCs have been shown to be a promising new therapeutic strategy for *Staphylococcus aureus* infections [116,172]. A subclass of ADCs is antibody–antibiotic conjugates (AACs). One of these, DSTA4637A, has been used for treating *S. aureus* infections, such as methicillin-resistant infections (Figure 5), achieving better results than antibiotics alone. For example, preclinical studies recorded better efficacy of DSTA4637A compared with vancomycin [173], and a Phase 1 clinical trial in normal volunteers established its safety and tolerability, as well as excellent pharmacokinetics. Despite current challenges, mAbs therapies have several advantages over antibiotics, including extended half-lives and a lower likelihood of cross-resistance. Recent developments in the design, manufacture, and delivery of mAbs for infectious diseases have substantially advanced this technology. For example, gene delivery devices such as adeno-associated virus vectors are future options for producing and delivering mAbs [174]. In fact, antibody engineering technologies have been also applied to enhance mAbs against other IOIs like SARS-CoV-2, respiratory syncytial virus (RSV), and Ebola [175]. Simultaneously, the COVID-19 outbreak is driving the translation of mAbs into the technology age, which may revolutionize the treatment of infectious diseases [176]. Nevertheless, there are still some hurdles to be cleared, including poor efficacy against some infections and very costly manufacturing [177]. For a mAbs stockpile (or global population) of all populations, manufacturing capability around the metric ton scale is needed [178]. Despite these headwinds, mAbs hold great potential to prevent new health threats and the rise in antibiotic-resistant bacteria [179,180].

### 4.5. Peptide- and Protein-Based Delivery Systems

Peptides and proteins are versatile biomolecules that not only act as therapeutic drugs by themselves but also as carriers for drug delivery. Their natural biocompatibility, biodegradability, and capacity to selectively bind to biological targets render them interesting candidates to overcome limitations related with traditional drug delivery systems. Peptide- and protein-based delivery systems are resulting in a new era in selective drug release, providing accuracy, efficiency, and biocompatibility.

Antimicrobial peptides (AMPs) are small, naturally occurring molecules exhibiting broad-spectrum antimicrobial properties. The peptides have the ability to trigger microbial membrane disruption, biofilm inhibition, and immunemodulatory activity. Delivery systems improve the stability and bioavailability of AMPs and target resistant pathogens [181]. AMPs interact with microbial membranes through electrostatic interactions to form pores and disrupt membranes. This occurs with a lower chance of developing resistance compared to traditional antibiotics [182]. AMPs also have the capacity to penetrate and disrupt biofilms, defensive groups for resistant bacteria [183]. Biofilms are one of the primary reasons for the challenge in treating long-term infections such as those caused by *Pseudomonas aeruginosa* [184]. AMPs also possess the capacity to modulate the host immune response, enhancing bacterial clearance and reducing inflammation. For instance, LL-37 is one of the potential drugs for the treatment of sepsis since it has a variety of characteristics, including inducing antimicrobial NETs and ectosome release, promoting programmed cell death (pyroptosis and NETosis) while retaining its bactericidal and LPS-neutralizing effects [185]. Meanwhile, therapeutic use of AMPs in combination with antimicrobials has also shown to be a promising regimen. This approach has the capability to overcome antibiotic resistance, improve bactericidal activities, and reduce toxicity and side effects. With greater compound selectivity, increased bacterial membrane permeability, and reduced antibiotic drug efflux, this strategy can effectively prevent bacteria [186]. Use of AMPs as therapeutics has been investigated in different configurations, such as direct injection, encapsulation in nanoparticles, and bio-dressings. For instance, Colistin, a cyclic AMP, is the last resort for treating Carbapenem-Resistant Enterobacteriaceae [187].

Protein-based nanoparticles (PNPs) are soluble, monodisperse, biocompatible, and have strong structures that make them promising among the nanocarrier systems. Proteins are natural biomolecules with different physicochemical characteristics that make them useful in nanocarrier systems. Firstly, their biocompatibility reduces the potential of adverse immune reactions, making them safer for therapeutic use [188]. Secondly, protein carriers are biodegradable and, on degradation, yield non-toxic amino acids, lowering systemic toxicity [189]. Thirdly, the hierarchical structure of proteins allows surface modification for drug delivery and release in a targeted and controlled fashion [190]. Finally, proteins allow high drug loading capacity and can accommodate hydrophilic and hydrophobic drugs in a beneficial way [191]. Albumin, transferrin, and ferritin proteins are among the most popularly used nanocarriers in drug delivery. The carrier has the potential to encapsulate AMPs or antibiotics, protect them against degradation, and target receptor-mediated endocytosis. Antibiotic-loaded albumin nanoparticles, for instance, exhibited improved activity against MDR Mycobacterium tuberculosis [192,193]. Secondly, peptide conjugation to gold nanoparticles (AuNPs) to frog skin AMP esculentin-1a, Esc (1–21), was determined to potentiate (15-fold) the free peptide against *Pseudomonas aeruginosa* with no cytotoxic activity on human cells [194]. Targeted delivery to infection sites is facilitated by fusion proteins that contain therapeutic peptides and targeting moieties. For this, an antibody–drug conjugate (ADC) was developed by conjugating an antimicrobial peptide to a specific monoclonal antibody (VSX) used against *P. aeruginosa*. It was therefore established that the ADC had strong killing activity against the bacteria with minimal cytotoxicity against mammalian cells and rescued mice from lung infection. Targeting ligands have also been conjugated to nanoparticles to target carbohydrate receptors on *Helicobacter pylori*. The lectin-conjugated gliadin nanoparticles have been effectively used for the treatment of *H. pylori* and are thus a good candidate for targeted drug delivery.

### 4.6. CRISPR-Cas Systems

CRISPR-Cas technologies have revolutionized genome editing and become a powerful tool for genome engineering due to their remarkable specificity and efficiency. Applications of CRISPR (Clustered Regularly Interspaced Short Palindromic Repeats) and Cas (CRISPR-associated) proteins have ranged from a bacterial and archaeal adaptive immune system to a powerful tool for genetic manipulation of organisms [195]. The saga of CRISPR-Cas systems is, in fact, a story many decades in the making, spanning various researchers across many countries and continents. This history is most effectively exemplified by the two female scientists who shared the 2020 Nobel Prize in Chemistry [196].

**Figure 6:**
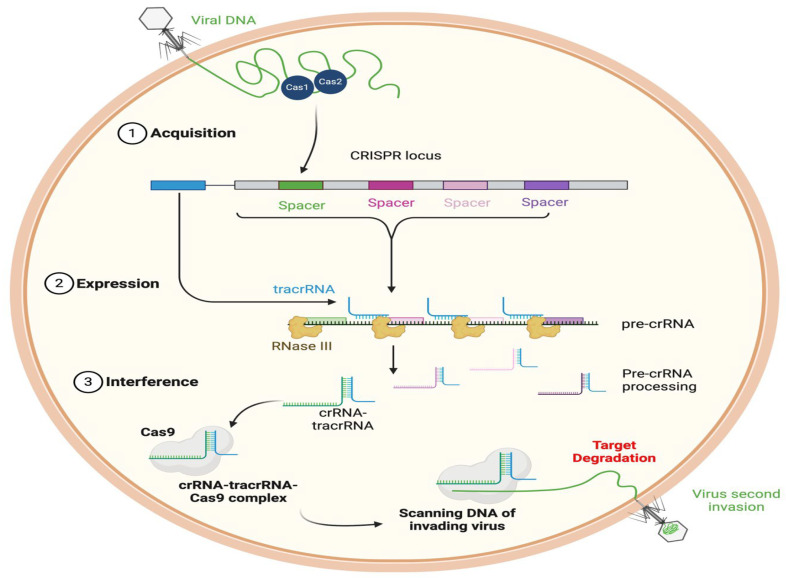
An overview of the basic mechanism of CRISPR-Cas gene editing system. The genome editing process involves the following steps: The first step is acquisition: when a bacterium is infected by a virus, it incorporates fragments of viral DNA (spacers) into its CRISPR locus as a memory of the infection. The second step is expression, where these spacers are transcribed into short RNA sequences (crRNAs), which guide the Cas proteins to target matching viral DNA during subsequent infections. The third step is interference; Cas proteins, guided by crRNA, cleave the viral DNA, preventing the virus from replicating (created with BioRender.com).

CRISPR-Cas9 is carried out in three stages (Figure 6). In the acquisition phase, the bacterium acquires fragments of the invading viral DNA, called spacers, and integrates them into its genome in the CRISPR locus. These spacers are a molecular memory archive of past infections. During the expression stage, the CRISPR locus is first transcribed as a pre-crRNA molecule and then cleaved into small crRNAs, each of which harbors a spacer that matches a segment of the viral genome. These crRNAs direct the system’s defense system. During the interference phase, the crRNA binds to the tracrRNA, and, together with a Cas protein, most often Cas9, the complex searches for complementary sequences to the crRNA in the genome of the attacking virus. Once a match is located near a protospacer-adjacent motif (PAM), a Cas protein cuts the viral DNA, rendering the attack ineffective [195,197]. And lastly, the newly added spacer is retained in the CRISPR locus and is effective for preventing future infection by the same virus. In this way, the bacterium is able to boost its defense system steadily [198]. This intricate machinery not only provides microbes with adaptive immunity but also serves as the basis for current genome editing techniques. The ability to construct a CRISPR-Cas9 method to recognize unique DNA sequences has transformed how research in genetics, medicine, and biology will be conducted.

CRISPR-Cas systems have several features to confront AMR in an exceptional manner. CRISPR-Cas can be programmed to cleave the antibiotic resistance genes in the bacterial genomes in a targeted manner. The system shuts down the genes by creating double-stranded breaks in them, making the bacteria susceptible to antibiotics once more. For example, in the specific case of resistance to the beta-lactams, “inactivation” of resistance determinants (such as the beta-lactamase genes that encode the beta-lactamases) “reactivates” the beta-lactam antibiotics. Alternatively, CRISPR-Cas can be modified to specifically kill resistant gene plasmids, limiting resistance spread throughout bacterial ecosystems. This practice is very effective in controlling MDR [199]. A catalytically dead Cas protein (dCas9) is used to silence transcription of resistance genes without inducing DNA cutting. By disrupting these genes, CRISPRi could suppress resistance in the short term, allowing the antibiotics to function more effectively [200]. Furthermore, CRISPR-Cas systems can be programmed to distinguish between pathogenic and non-pathogenic bacterial strains according to their specific genetic patterns. Such selective targeting is exploited to maintain the beneficial flora intact and kill only the dangerous pathological threats [201]. Cas13 enzymes function by targeting RNA, not DNA, and it is possible to directly kill messenger RNAs for resistance or virulence factors. This strategy offers a temporary but powerful means of regulating bacterial pathogenicity [202]. The method presented by Wang et al. is ATTACK, an alternative to the treatment of MDR bio-pathogens using combined Associative TA (toxin and antitoxin) and CRISPR-Cas. ATTACK is a reverse acronym for “Associates Toxin-Antitoxin Systems and CRISPR-Cas to Kill MDR Pathogens”. It exploits the inherent TA systems in bacteria. A combination with the CRISPR-Cas mechanism adds specificity and efficiency to the latter, allowing to efficiently eliminate pathogens that acquired drug resistance [203]. TA systems consist of a pair of genes, with one gene encoding a toxin, which is capable of killing or arresting growth of bacteria, and the second gene encoding an antitoxin, which neutralizes the toxin. In the ATTACK strategy CRISPR-Cas is employed to knock down the gene for the antitoxin, which liberates the lethal killer effect upon the specific bacteria [204]. CRISPR-Cas is specifically designed to locate a predetermined genetic target for MDR strains. This only activates the TA system in the target pathogen and hardly damages commensal and non-pathogenic bacteria [205].

## 5. Challenges and Limitations

### 5.1. Biological Barriers

One of the potential obstacles in designing and developing alternative nanoparticle-based antimicrobials is associated with their complex interactions with bacterial cells. These interactions are controlled by both the diversity of nanoparticles and bacteria but also with the capacity of bacteria to regulate their physicochemical properties at the cellular level, including active responses caused by nanoparticles such as the change in surface charge [206,207] or biofilm matrices that mediate nanoparticle aggregation [206,208,209] and even induce efflux pumps for toxic metal ion removal [210]. They may also accumulate or enzymatically detoxify metal ions as a protective response [206,211]. These resistance mechanisms are often controlled by operons or plasmids that can be transferred horizontally, particularly during sub-lethal exposure to nanoparticles [206,212]. In addition, the challenge in a thick bacterial biofilm is that the microenvironment created includes an acidic pH, hypoxia, special enzyme activity, and a high concentration of hydrogen peroxide, all of which severely reduce the efficacy of nanoparticles via ROS production [213,214].

Additionally, while the characteristics of stimuli-responsive nanoparticles enhance biofilm penetration, their localization at infection sites is only modest compared to whole-body dosing. After intravenous administration, a high quantity of nanoparticles accumulates in the spleen and liver, which may cause damage to health; thus, there is a requirement for extensive studies regarding chronic toxicity and the effect on the reproductive system of nanoparticles [215,216,217]. Alongside nanoparticle-specific problems, aspects of the NP itself may be seen as foreign by the immune system leading to immune reaction. Strategies which can be used for preventing or reversing an established immune response are the establishment of immunological tolerance, changes in the formulation/product and control over immune responses using small-molecule drugs, and manipulating lymphocytes to block the immune response [218,219]. Examples of such changes are PEGylation, which enhances stability and circulation of drugs, as observed in Doxil^®^ and mRNA COVID-19 vaccines [218,219], and encapsulation, which confers protection to the drug and modulates its release, e.g., paclitaxel-loaded nanoparticles and chitosan-coated capsules for proteins [218,219]. Furthermore, and equally importantly, is the high level of unpredictability in the behavior of nanoparticles inside biological fluids in vivo. In blood, they quickly adsorb to serum proteins, thereby forming a protein corona, which strongly affects their characteristics. This layer may improve their targeting or alternatively not yet identified sites and reduce specificity [220,221,222,223]. Thus, it is important to elucidate and manage the protein corona formation process for enhancing the performance of NP-based delivery systems with predictable clinical efficacy [221]. Finally, major drawbacks of peptide- and protein-based delivery systems are their fragility leading to degradation because they are easily denatured breaking down via proteolytic enzymes (including gastrointestinal enzymes) when administered orally [218,219]. In addition to these limitations regarding degradation, the unpredictable biological behavior of NPs provides an additional level of complexity in translational research. For instance, one’s gut microbiome has a strong impact on drug metabolism in the body as bacteria can modify drugs before entering the bloodstream by either activating or degrading them [224,225,226]. This occurs erratically with diet, age, and antibiotic use, additionally leading to marked interindividual variability, making dose standardization a challenge and leading to variable both efficacy and toxicity [225].

### 5.2. Stability and Scalability

Manufacturing methods in nanomedicine frequently face variability issues, which undermine large-scale production and the transition to clinical applications. To tackle these challenges effectively, implementing standardized processes and quality control measures is essential [227]. Furthermore, the high production costs associated with the large-scale production of protein-based antimicrobials can be prohibitive, thus restricting their accessibility. This can be modified by utilizing plant platforms for the synthesis of AMPs, as opposed to the costly solid-phase peptide synthesis (SPPS) and microbial bioreactors, which produce hazardous environmental waste [228]. Plant-based systems present a viable alternative, reducing both upstream and downstream production expenditures, simplifying scale-up processes [228,229,230] and advancing environmental sustainability through diminished energy consumption and negligible toxic waste generation [228,229]. In a study conducted by Özakar et al., the stability of boron nitride nanoparticles was explored. The nanoparticles were stored in darkness at room temperature (25 ± 2 °C) and moderate humidity (60 ± 5%) for 90 days. The samples were reassessed for zeta potential, polydispersity index (PDI), and particle size and were then compared to freshly prepared formulations. The findings revealed a statistically insignificant change (*p* > 0.05) in colloidal stability after 24 h. However, a relatively significant change (*p* < 0.05) was observed for measurements taken on the 30th and 90th days. This suggests that while boron nitride NPs are initially stable, their long-term use may necessitate protective formulations or storage modifications [231]. In addition to the issues regarding conventional nanoparticles, specific configurations with magnetic nanoparticle (MNP)-based drug delivery face further challenges, and these have been reviewed by Yue Zhuo et al. These systems encounter significant hurdles, driven mainly by the BBB and the immune system. Despite being able to mediate targeted drug delivery across the BBB, the dimension and surface properties of MNPs need to be well-controlled for secure, effective, and quality treatment [232]. In conclusion, these challenges highlight the complexity of translating nanomedicines from the laboratory to clinical practice.

### 5.3. Regulatory and Ethical Challenges

The regulation of nanomedicines by agencies such as the U.S. FDA and the European Medicines Agency (EMA) is executed on a case-by-case basis, utilizing the conventional framework for evaluating benefits against risks. Notwithstanding this structured approach, it is frequently observed that such assessments advance in the absence of universally standardized or harmonized regulatory criteria specifically tailored to this category of therapeutics. The FDA regulates pharmaceutical products under two primary statutes: the Federal Food, Drug, and Cosmetic Act (FDCA), which pertains to chemically synthesized drugs and medical devices, and the Public Health Service Act (PHSA), which governs biologically derived therapeutic agents, so the classification of these products is based on their modes of action, which can be categorized as chemical, mechanical, or biological [233,234,235]. For instance, mechanical action might be exemplified by drug-eluting stents, whereas biological action is more applicable to protein-based therapies [233,234,235]. Nanomedicine formulations intended for clinical use need to adhere to the regulatory pathways of that country to ensure efficacy and safety. This may occur under one of the following submission types if applicable: Investigational New Drug (IND); New Drug Application (NDA); Abbreviated New Drug Application (ANDA); or Biologics License Application (BLA) [234]. Because they are multi-component drug products, nanomedicines require extensive characterization at all steps during development and in regulatory submissions [234]. The FDA has not yet published rules specifically tailored for nanomedicine; however, progress is being made in this regard. Especially, the FDA assesses nanomedicines based on their unique physical and chemical characteristics as well as biological properties rather than simply evaluating their safety and effectiveness only [234,235]. The FDA’s Guidance for Industry emphasizes two main factors: whether a completed good or material is purposefully engineered to have at least one dimension or structural feature within the nanoscale range (about 1–100 nm) and whether the product is designed to show properties or physical, chemical, or biological effects arising especially from its nanoscale features [236]. These considerations enable regulators to identify which products require a higher level of scrutiny due to their unique nanoscale behavior.

Another challenge for NPs is the ethical concerns related to their medical applications, which involve regulatory definitions that establish criteria for nanotechnology. Because of their complex and until recently unprecedented nature, patients struggle to understand the potential risks and long-term effects, so informed consent becomes the primary ethical challenge in nanomedicine [237,238]. Indeed, when it comes to confidentiality or equity of access to information, there are significant ethical and practical considerations associated with the implementation of nano-enabled therapies. These therapies are associated with high production costs and frequently involve delicate genetic information, which is associated with concerns on the confidentiality of patient identities and potential for medical inequalities [238,239]. Thus, more than just technological advances are required to overcome these barriers; access must be ensured for everyone, equitable policies must be adopted, strong information security systems are required, and the potential benefits of NPs in health, research, and clinical systems need to be fairly distributed [238]. In the ORBIT-3/4 studies of inhaled liposomal ciprofloxacin, patients provided informed consent according to international ethical codes [240]. The cost of this therapy and whether it will be accessible to the public are unknown; however, there are underlying questions surrounding equity in access to technologies that become available for purchase on a marketplace. Thus, the challenges posed by these ethical and regulatory considerations are an important barrier to overcome between discovery in the laboratory and implementation in practice. Table 3 summarizes the pros and cons of each delivery platform, underpinning the mechanisms of AMR with their respective targeted delivery platforms. It contains several promising studies of experiments using different TDD systems for infection treatment and diagnosis.

## 6. Future Directions

### 6.1. Emerging Materials and Technologies

#### 6.1.1. Biomaterials

The unique physicochemical properties of nanoparticles and their flexibility make nanoparticles an ideal choice to release the drug and control it. Several strategies can be engaged internally such as enzymes, pH, and redox stimuli to activate the maximum response of nanomaterials. Within this specific milieu, those intelligent nanocarriers go through multiple reactions physically and chemically like hydrolytic cleavage, protonation, and molecular conformation modification to release the targeted drug [39]. For example, abnormal pH, reactive oxygen species (ROS) and redox in the tumor case can act on intranuclear endosome/lysosome escape, which controls the drug release and activates the drug precursors; these targeted intracellular stimuli help to treat specific tumor [252]. Changing the internal pH level by modifying the ester, hydrazine, and acetal group on the nanoparticle surface is effective in the release of drugs, such as cRGD-Dex-DOX/HDZ, which had the fewest side effects over all other controls and showed the most prominent antitumor effect [253]. Applying P-LDH improved cellular uptake and had high drug-loading [254] and Gd-DTPA/CaP, which efficiently killed tumor cells without harming the surrounding healthy area [255]. Nap-FFGPLGLARKRK effectively suppressed tumor growth and metastasis and limited adverse effects [256]. TPD&FPD&D micelles improved the antitumor role and increase the cytotoxicity of the drug [257]. By employing these properties, smart NPs are developed, which decrease the bacterial resistance by delivering the drugs to the site of infection. Experimentally, NPs protect the antibiotic from degradation, provide a high uptake of the drug by microorganisms through various routes rather than the free drug, and increase the stability of NPs’ function. Regular cross-linking is also necessary; variations in purity are considered a limitation [258]. In addition, the application of hybrid nano-system approaches is an efficient way to improve biocompatibility and achieve maximum delivery and targeting.

A polymeric kernel and lipid or lipid-PEG covers the core shell of the nanoparticle. This modification improves the biocompatibility and physical stability between polymeric nanoparticles and liposomes. PLN hybridization demonstrated a promising nanocarrier for biomedical imaging and drug delivery, marking a significant development in nanomedicine. Various types of these nanocarrier polymers, including synthetic, semi-synthetic, and natural, offer excellent stability, are easy to prepare, and are seamless. In addition, they self-assembled into a sustainable and predictable pattern when the nanoprecipitation technique was applied, making them significantly scalable [259]. Nanoprecipitation is a popular technique used to create a polymer core smaller than 100 nanometers that covers both a drug and polymer. Stable and weak polymers are used, with the former dissolving before the latter. After that, the polymers precipitate and separate when complete miscibility of both phases is finished. For example, a drug for polymeric lipid hybrid nanoparticles, which are made of a combination of lipid or liposomes with polymer particles, permits NP formulation to be repeatable and scalable for experimental applications [259].

Poly (lactic-co-glycolic acid) (PLGA) is characterized as a biocompatible and biodegradable polymer approved by the FDA, and it is primarily used for drug delivery. It is hydrolyzed into small units of glycolic acid and lactic acid. It is utilized by the citric acid cycle within the body. Some features affect its mechanical strength, such as size, biocompatibility, ability to release the drug, and capacity for entrapment. Additionally, its minimal toxicity makes it suitable for biomedical applications and drug delivery. These copolymers are commercially available in various sizes and molecular weights, depending on the copolymer ratio and degradation times. One of the significant biological barriers of this combination of PLGA-based NPs is the recognition process by the body, whereby the hydrophobic particles are identified as foreign molecules due to their particle surfaces being covered with a hydrophilic layer. So, opsonin proteins recognize specific particles, attach to them, and initiate phagocytosis. Polyethylene glycol (PEG) is a hydrophilic, non-ionic, biocompatible polymer that facilitates surface modification for chemical moieties. PEG can also combine with Chitosan, poloxamines, and poloxamer, which alter the surface by inhibiting hydrophobic and electrostatic interactions, thereby increasing its half-life during blood circulation. Doxorubicin, gentamicin, and sparfloxacin are the ideal candidate antibiotics for this modification. Recently, researchers have focused on enhancing effectiveness and reducing the risk of side effects by loading PLGA-based NPs with antimicrobial agents, which helps increase the release and achieve the target accuracy after several in vivo and in vitro evaluations. This step makes PLGA-based NPs a potential candidate for inhibiting bacterial growth and enhancing the drug’s physicochemical properties [258].

Liposomes are biodegradable and nontoxic concentric phospholipid bilayers found as spherical vesicles with an aqueous core. Commonly used in drug delivery systems, they can stabilize compounds, thereby enhancing the therapeutic efficacy of the drug. Many factors determine the ideal technique for liposomal drug encapsulation, including the liposome stability, drug-to-lipid ratio, encapsulation efficiency, drug retention and leakage, sterility, scalability, production efficiency, and cost efficiency [260]. To manipulate a new liposome in vivo, drug toxicity and behavior should be considered. It takes more than two years to form a liposomal drug product because it is less stable in a colloidal system. So, it is stored in lyophilized form to enhance stability [261]. Currently, most liposome formulations are undergoing clinical trials to predict their safety within cells and require further development of patterns and methods for therapeutic applications. Conversely, solid lipid nanoparticles (SLNs) have one or more solid saturated fatty acids and are common within the first generation of lipid nanoparticles [262]. SLNs have a significant physicochemical stability and are commercially lyophilized and sterilized in a cost-effective way [92].

Metal-based nanoparticles are among the most common inorganic nanoparticles and are used to combat the antimicrobial resistance associated with traditional antibiotics. Silver, gold, zinc oxide, copper, and copper oxide nanoparticles do not bind to the specific receptors of the microorganism cell, which reduces bacterial resistance and maximizes antibacterial activity. Their applications are recorded with Gram-negative and Gram-positive bacteria. Size, surface energy, roughness, and shape are specific features that affect their function. As a result, positive-charge nanoparticles bind to the negative charge of the cell due to electrostatic interactions, leading to the destruction of the cell wall and an increase in permeability. Moreover, metal ions released from NPs enter the cell and then destroy it. Sánchez-López et al. reported that AgNPs were highly reactive and exhibited high antibacterial activity compared to other nanoparticles (e.g., AuNPs, ZnONPs, and CuONPs), which may accumulate and increase the toxicity to body organs [263]. The toxicity effect of metal NPs is influenced by several parameters such as size, concentration, shape, and surface coating, which elevate the cytotoxicity of the living cells and enhance apoptosis and necrosis [254]. When comparing the cytotoxicity level between negative and positively charged AuNPs, Huhn et al. reported that the positive NPs were more toxic than the negative ones due to the increase in their uptake [264], and on the other hand, AgNPs have higher potential toxicity than AuNPs. One of the best strategies to overcome the potential toxicity of NPs is surface modification, which is reversible by non-covalent modification [265]. Patlolla et al. coated AuNP surfaces with PEG and reported a less hepatotoxic effect than that of uncoated AuNPs in Sprague Dawley rats, so when AuNPs are functionalized by PEG, it easily to bind the cell membranes and increase the penetration to target cells and reduce the toxicity of AuNPs. Also controlling the dose of metal NPs can conserve the cells. Furthermore, AgNPs at lower concentrations have been found to be relatively safe for human cell lines and exhibit minimal cytotoxic effects [266]. Further studies are recommended to approve these metals and elucidate their behavior in vivo, thereby facilitating pharmaceutical development [263].

Polymers can hold water through three-dimensional cross-linked molecular networks, known as hydrogels, which are formed by covalent and noncovalent bonds. These polymers can be manipulated to meet the requirements of use in biomedicine, tissue engineering, and biosensors, including adhesiveness, anti-adhesion, shape-memory, toughness, conductivity, elasticity, stretchability, strength, and self-healing ability [267]. Nanocomposite (NC) hydrogels are an ideal material for enhancing the bioavailability and therapeutic efficacy of medications as they enable targeted or controlled drug release. Common examples are locust bean gum (LBG), poly (4-acryloylmorpholine) (PAcM), and silver nanoparticles (AgNPs) [268]. To integrate NPs into the hydrogel network, NPs can work as fillers in the hydrogels without involvement in the network or blending NP formation. For example, in drug delivery, AuNPs with NC hydrogels can be used for wound dressing, and AgNPs with antibacterial properties can also be employed. Moreover, to control the drug release, magnetic response of iron oxide NPs is applied to heat NC hydrogels. Therefore, NC hydrogels effectively provide suitable functions and enhance mechanical features compared to traditional approaches [269].

Poly (amidoamine) (PAMAM) dendrimers are widely used in nanomedicine due to their well-defined, branched structure and biocompatibility. They act like nanoparticles because they contain tertiary amine groups at their branching points. These amine groups can attract or bind metal ions once dissolved in water. The metal ions, after coordination, can also be chemically reduced to the neutral (0) state in the dendrimer and form small nanoparticles confined within the dendrimer scaffolds [270]. This feature gives PAMAM dendrimers a wide variety of options to mimic bio-molecules, including viruses, proteins, and enzymes. They improve the solubility, stability, and bioavailability of poorly soluble drugs and enhance the targeting of drugs to specific tissues by conjugation with ligands and the penetration of cells and cell membranes by use of a uniform size of carrier. Another class of dendritic polymers is the poly (L-lysine) (PPL) dendrimers, which are commonly used in antibacterial work. When the surface is modified by adding tertiary alkylammonium groups, they become very effective antibacterial agents. In addition, chitosan–PPL hybrids have shown effectiveness in this role [271]. These surface modifications increase the targeting efficiency and sensitivity of drug delivery systems. Other common modifications include PEGylation, glycosylation, acetylation, and amino acid functionalization, which serve to neutralize the outer amine groups and enhance biocompatibility. PPL dendrimers are ideal for drug delivery because of their well-defined shape, size, branching pattern, and internal cavity. Furthermore, they can be used to attach imaging molecules or targeting ligands, offering multifunctional capabilities in modern nanomedicine [272].

#### 6.1.2. Advanced Multifunctional Hybrid System

Hybrid conjugates, both inorganic and organic, combine the proprieties of two or more materials. They include hydrogel, magnetic NP, ceramics, carbon nanotubes, and natural polymers. This system is used to overcome the instability and poor biocompatibility of nanoparticles. Hydrogels exhibit excellent biocompatibility and remain localized at the administration site, but their ability to regulate drug release is relatively weak. Conversely, a few remaining NPs exit the inaction site and show divertive biocompatibility yet can offer precise modulation and material release as a drug [273]. Khalid et al. conducted a study to formulate and characterize hydroxypropyl-o-β-cyclodextrin (HPβCD) hybrid nanogels for solubility enhancement of the lipophilic drug dexibuprofen. They found that highly porous and amorphous nanogels showed significant dexibuprofen release in aqueous medium, which revealed efficient solubilization of the drug by HPβCD hybrid nanogels. The study was confirmed by FTIR, TGA, and DSC studies. Furthermore, in toxicity studies, no significant changes in behavioral, physiological, biochemical, or histopathological parameters of animals endorsed that developed formulations are nontoxic and biocompatible [274].

Carbon quantum dots (CQDs) and carbon 2D nanosheets (graphene oxide, graphene, and graphdiyne) showed remarkable physicochemical attributes in antimicrobial claims and drug delivery. CQDs are characterized by high surface area and tunable porosity, which enhance tissue generation and affect drug release, with encouraging results in promoting treatment outcomes and decreasing the systemic toxicity effect [275]. Liang et al. developed CQDs to target delivery of doxorubicin to breast cancer cells, and they observed improvement in the treatment efficacy with reduced adverse effects [276]. For the optimum efficiency in the delivery and the therapeutic effect, CQDs and NPs of liposomes were used by Zhu et al. [33]. In addition, the fundamental reason for the toxicity of CQDs has not been entirely elucidated, and further investigations are necessary to evaluate their cytotoxicity with other cells [277].

The efficacy of antibody-functionalized PLGA nanoparticles, prepared by nanoprecipitation, increases the bioavailability and potency of current antibiotics. Several studies have reported the functionalization of NPs with monoclonal antibodies, which illustrate the functionalization and application of Au/Ag nanoparticles with anti-MRSA antibodies in murine models suffering from pneumonic complications specific to MRSA infection. The results showed an inhibition of bacterial growth in vivo. Another example is anti-protein A antibody-functionalized nanoparticles, which are used to selectively eliminate *Staphylococcus aureus*. This application is designed to manage non-healing wound areas, either by using photothermal therapy or a combination of it with appropriate antibiotics. The use of these metal nanoparticles functionalized with antibacterial agents and photosensitizers for photodynamic therapy increases the selectivity for the bacteria when co-cultured with eukaryotic cells. The anti-protein A antibody-modified PLGA-based NPs were also developed as drug-loaded carriers to deliver rifampicin for the targeted and effective treatment in the murine model. To sum up, the difference between using metal or inorganic nanoparticles is the capability of those polymers to release their encapsulated antimicrobial in a controlled and regulated pattern in addition to chemical and physical factors, the flexibility of their surfaces for functionalization, biodegradation, nature of polymers, and the method of application [193].

Metal–organic frameworks (MOFs) have specific structures, high porosity, extensive surface areas, highly effective nucleus-targeted delivery, and antimicrobial uses. Composite coatings combining MOFs with sulfonated hyaluronic acid on medical implants enhance compatibility with biological tissues and suppress bacterial growth. Simultaneously, metal and metal oxide NPs employ multifaceted antibacterial mechanisms, lowering the risk of resistance development. When paired with antimicrobial agents, these NPs create hybrid systems not only boost antibacterial effectiveness but also help reinstate the potency of existing antibiotics. This strategy offers several benefits, including reduced therapeutic doses, less dose-related toxicity, slower resistance development, and shorter treatment times [278].

### 6.2. Personalized Medicine Approaches

Personalized medicine is increasingly important in overcoming AMR by adapting the pharmaceutical delivery system to individual patient profiles. Advances in the sequencing of the genome and genomics have facilitated the discovery of the particular resistant genes in pathogens and have also contributed to directing the therapies to be more specific, effective, and less prone to fueling resistance [279,280,281]. For example, the UK 100,000 Genome Project has demonstrated that bringing genomics together with clinical information has the potential to inform the choice of antibiotics based on resistance and with greater precision in therapy [282]. In addition, personalized treatment has been successful in the management of tuberculosis, with line probes and WGS helping to tailor second-line drugs in cases of multidrug-resistant tuberculosis [283]. New technologies such as 3D printing, organ-on-chip platforms, and microfluidics have revolutionized the production of specific patient drug delivery systems. These developments in the control of drug release mode, shape, and dose in the target region significantly improve on therapeutic efficiency and minimize the systemic toxicity [284,285]. Furthermore, artificial intelligence and machine learning algorithms are also leveraged for predicting the patients’ response to antibiotics, selecting the best compositions of drugs, and simulating the outcomes of the treatments. AI-driven platforms such as DeepARG and PathoFact now enable doctors to predict resistance phenomena in metagenomic data and to recommend targeted therapies in real time [286,287]. Furthermore, pharmacogenomics has gained ground in modifying drug metabolism and minimizing adverse reactions, especially for drugs with narrow therapeutic windows or high toxicity such as aminoglycosides [288]. Integrating these technologies into clinical practice not only improves the outcome of treatment and reduces the prevalence of multidrug-resistant infections but also optimizes the use of existing antimicrobials, minimizing experimental therapy and drug misuse.

Recent trends also mirror the emerging significance of intellectual property (patents) and clinical trial efforts in speeding up antimicrobial resistance (AMR) research. The United States Patent and Trademark Office (USPTO) patent database register is steadily increasing patent technologies to combat AMR, such as antimicrobial peptides, bacteriophages, and CRISPR antimicrobials [289,290,291,292]. Likewise, clinical trial records from international registries substantiate the clinical translation of such innovation. Phase III clinical trials that are underway involve the use of bacteriophages to compete with multidrug-resistant *Pseudomonas aeruginosa*, CRISPR-Cas3-containing antimicrobials to address resistant *Escherichia coli*, as well as new β-lactamase inhibitors to address carbapenem-resistant Enterobacteriaceae [293]. Besides this, nanotechnology-enabled antimicrobial formulations, such as liposomal amikacin as well as treatments of wounds with silver nanoparticles, progressed to Phase II as well as Phase III clinical trials, demonstrating their clinical promise [293]. Accordingly, patent activity trends as well as clinical trial advances represent a rich innovation pipeline within AMR research connecting discovery with patient-centric therapeutic use.

### 6.3. Interdisciplinary Collaboration

#### Microbiology, Materials, and Pharmacology Come Together

A combined and interdisciplinary effort is necessary to fight AMR by merging microbiology, materials science, and pharmacology. Microbiologists contribute to the ecology of resistance, the factors associated with infection, and the physiology of microbial organisms, allowing the therapeutic targeting of specific pathogenic pathways. An excellent example is cationic AMPs, designed from host-defense microbiology screening and confirmed with pharmacological model [294]. Moreover, material scientists take the lead in stimulating responsive drug delivery systems by mouth, in which pH-sensitive or enzyme nanoparticles deliver antibiotics under specific infectious conditions to improve local effects and reduce side effects [81,123]. Physicists also realized a black silicon nano-sci-shaped surface whose bactericidal effect on Gram-positive and Gram-negative bacteria was demonstrated by microbiologists as based on non-antibiotic-dependent mechanical failure of bacterial cells [295]. Pharmacologists refine the principles of absorption, distribution, metabolism, and elimination of drugs (ADME) such that the window between therapeutic effect and toxic effect is maximized. For instance, vancomycin encapsulated in liposomes improved drug delivery to MRSA-infected tissues while attenuating renal toxicity, again reflecting close interactions between drug formulation and clinical microbiology teams [296]. Interdisciplinary efforts also lead to tools like the nanocarrier CRISPR-Cas delivery platform, which disables certain resistance genes in bacteria. These will be complemented by research on a genetic level and nanotechnology, representing the possibility of reversing rather than just winning the race against resistance [297,298].

## 7. Conclusions

AMR has become one of the major potentially unsustainable global health threats, favored by evolutionary adaptation at both genetic and biochemical levels that eventually leads to therapeutic failure. Since the development of new antibiotics is lagging behind the emergence of resistance mechanisms, novel approaches are urgently required. Targeted drug delivery represents a promising shift toward decreasing systemic toxicity and improving antimicrobial potency of therapeutics through a targeted delivery and localization as compared to conventional delivery.

Recent advances in drug delivery technologies have emphasized various approaches, such as nanoparticle-based drug carriers, stimuli-responsive delivery systems, bacteriophage-delivered medicine, antibody–drug conjugates, peptide/protein-based drug carriers, and CRISPR-Cas platforms. Both ways have distinct applicative potential in overcoming resistance mechanisms, increasing drug stability, and thereby recovering the effectiveness of current antimicrobials. Yet obstacles like biological barriers, scaling, regulation, and ethical concerns must be overcome before their broader clinical application becomes feasible.

Thus, future fusion of these next-generation materials and smart technologies with site-specific delivery devices may revolutionize the therapeutic approaches. Personalized medicine, through individualizing interventions based on patient-specific microbiomes (anthropological bodies of humans) and resistance profiles, is necessary to realize the full potential of antimicrobials to maximize efficacy with minimized nontarget effects on beneficial microbiota. In addition, cross-discipline teamwork between microbiologists, clinicians, nanotechnologists, as well as policymakers is expected to be decisive to expediting improvement in translation terms.

In conclusion, whilst AMR is a fast-moving and urgent risk, the opportunity for innovation through targeted drug delivery is significant. New technologies in combination with targeted therapeutic approaches may be adopted to enhance the utility of antimicrobials and improve the response to resistant infections globally.

## Figures and Tables

**Table 2:** Comparison of stimuli-responsive drug delivery systems.

Stimulus Type	Trigger Mechanism	Target Environment	Example/Application
**pH-responsive**	Structural change or degradation at lower pH	Acidic sites such as inflammatory zones, tumors, biofilms, and oral cavities	Treatment of *A. baumannii* infection; prevention of gingivitis and cavities [158,159].
**Temperature-responsive**	Drug release triggered by local or external temperature increase	Inflammatory sites (local heat) or externally heated areas	Heat-induced drug release at specific sites [156].
**Enzyme-responsive**	Activation via enzymes overexpressed in infection (e.g., bacterial lipases)	Infected tissues with high enzyme expression	Lipase-sensitive delivery for Gram-negative and Gram-positive bacterial infections [75].

**Table 3:** Comparative analysis of targeting strategies.

Targeted Drug Delivery (TDD)	Advantages	Disadvantages	TDD and Its AMR Mechanism	Examples of Treatment/Diagnosis of Infections
Nanoparticle-based systems	Site targeted delivery High loading stability Biocompatibility Rapid AMP release	Complex synthesis procedures. Rapid clearance by the immune system. Aggregation and degradation	Penetrate membrane and target DNA/enzymes/metabolism, alter permeability, alter adhesion and inhibit biofilm	Colistin-loaded liposomes (lower systemic toxicity and higher survival rate of mice infected with *Pseudomonas aeruginosa* [136,137]
Stimuli-responsive systems	Targeted release at acidic pH, elevated temperature, or enzyme-rich sites. Stable at physiological pH	Complex synthesis, poor reproducibility. The pH/temperature difference between tumor and normal tissue may be too small for precise control	pH-responsive carriers disintegrate and degrade in the acidic microenvironment (inflammation, tumors, biofilms) on site and release the enzyme-responsive (lipase-sensitive) drug where bacterial enzymes are present	pH-responsive carriers for *A. baumannii* infection control [159]
Bacteriophage-Based Delivery	Synergy with antibiotics Fewer phage/antibiotic resistance mutants Can carry drugs/NPs/genes. Can re-sensitize resistant bacteria	Needs further safety profiling and clinical standards	Direct bactericidal lysis phage-derived enzymes degrade bacterial polysaccharides, leading to improved antibiotic penetration. Combined thereby activates biofilm destruction and bacterial eradication	Phage + daptomycin markedly increase killing of *E. faecium* and decrease resistant mutants [161] Re-sensitization of colistin resistance after phage exposure [161]. Phages act as delivering tools for genes/antibiotic/NPs [77]
Antibody–Drug Conjugates	Pathogen-specific targeting (protecting host microbiota), reduced potential for resistance High antibacterial activity Extended half-lives Activity against Gram-positive bacteria and *S. aureus*	Poor efficiency for some infections, very costly manufacturing, large scale capacity needed	Antibody-guided delivery of chemotherapeutic/antibiotics In some cases DSTA4637 outperforms antibiotics in preclinical studies. Gene delivery vectors proposed for mAb production/delivery	DSTA4637 against *S. aureus* was better than vancomycin [163,173] Effective antibodies against *Clostridium difficile* [169]
Peptide- and Protein-Based Delivery Systems	Natural biocompatibility Low toxicity enables broad-spectrum activity High target specificity effectively combating biofilms and reducing resistance risk when used with antibiotics and nanocarriers.	Short half-life, environmental sensitivity, low bioavailability, potential immune reactions, high production costs, and the need for extensive safety testing	Antimicrobial peptides (AMPs) bypass bacterial defenses by targeting membranes, creating pores, inhibiting efflux pumps, disrupting biofilms, and remaining effective against enzyme alteration	LL-37 for skin and soft tissue infections caused by MRSA and *S. pneumoniae* [241,242]. Lactoferrin has a promising efficacy for respiratory tract infections caused by many viruses, including SARS-CoV-2 [243]. Plectasin as a treatment for abdominal infections caused by *Streptococci* [244]. CP10A is a derivative of Indolicidin and has antimicrobial activity against *S. epidermidis* for the prevention of prosthetic device infections and biofilms [245]
CRISPR-Cas Systems	The tool efficiently targets specific DNA or RNA in various pathogens, eliminates antibiotic resistance genes, minimizes microbiome disruption, and can be easily adapted for emerging threats	Off-target cleavage may occur due to design errors and delivering CRISPR to infection sites. Limited efficacy in systemic infections, potential immune reactions, microbial resistance development, and biosafety and ethical concerns	CRISPR gene editing can target resistance genes, disrupt mutated drug targets, reduce drug efflux, and enhance antibiotic entry	CRISPR-Cas9 antimicrobials can be potentially used for skin infections caused by *S. aureus* [246]. Very promising in treatment of MRSA by targeting resistant genes like *mecA*, *aacA*, and *grlA* and *grlB* [247]. Eliminating Gram-negative bacteria from mixed cultures like *E. coli* and *S. enterica* through targeting chromosomal genes essential for metabolism and cell division [248] Treatment and diagnosis of MDR *E. coli* by targeting carbapenem and colistin resistant genes [249]. Potential treatment of HIV virus by excision of HIV-1 DNA from the genomes of infected people [250]. Potential treatment of HPV virus by disrupting the *HPV16-E7* gene with the which can trigger apoptosis and inhibiting the growth of HPV16-positive cervical cancer cells [251]

## Data Availability

No new data were created or analyzed in this study.
